# Polyanhydride nanovaccine against H3N2 influenza A virus generates mucosal resident and systemic immunity promoting protection

**DOI:** 10.1038/s41541-024-00883-3

**Published:** 2024-05-31

**Authors:** Christopher E. Lopez, Zeb R. Zacharias, Kathleen A. Ross, Balaji Narasimhan, Thomas J. Waldschmidt, Kevin L. Legge

**Affiliations:** 1 Department of Microbiology and Immunology, University of Iowa, Iowa City, IA USA; 2 Interdisciplinary Immunology Graduate Program, Department of Pathology, University of Iowa, Iowa City, IA USA; 3 Nanovaccine Institute, Iowa State University, Ames, IA USA; 4 Department of Chemical and Biological Engineering, Iowa State University, Ames, IA USA

**Keywords:** Protein vaccines, Antibodies, Germinal centres, Immunological memory, Influenza virus

## Abstract

Influenza A virus (IAV) causes significant morbidity and mortality worldwide due to seasonal epidemics and periodic pandemics. The antigenic drift/shift of IAV continually gives rise to new strains and subtypes, aiding IAV in circumventing previously established immunity. As a result, there has been substantial interest in developing a broadly protective IAV vaccine that induces, durable immunity against multiple IAVs. Previously, a polyanhydride nanoparticle-based vaccine or nanovaccine (IAV-nanovax) encapsulating H1N1 IAV antigens was reported, which induced pulmonary B and T cell immunity and resulted in cross-strain protection against IAV. A key feature of IAV-nanovax is its ability to easily incorporate diverse proteins/payloads, potentially increasing its ability to provide broad protection against IAV and/or other pathogens. Due to human susceptibility to both H1N1 and H3N2 IAV, several H3N2 nanovaccines were formulated herein with multiple IAV antigens to examine the “plug-and-play” nature of the polyanhydride nanovaccine platform and determine their ability to induce humoral and cellular immunity and broad-based protection similar to IAV-nanovax. The H3N2-based IAV nanovaccine formulations induced systemic and mucosal B cell responses which were associated with antigen-specific antibodies. Additionally, systemic and lung-tissue resident CD4 and CD8 T cell responses were enhanced post-vaccination. These immune responses corresponded with protection against both homologous and heterosubtypic IAV infection. Overall, these results demonstrate the plug-and-play nature of the polyanhydride nanovaccine platform and its ability to generate immunity and protection against IAV utilizing diverse antigenic payloads.

## Introduction

Influenza A virus (IAV) causes seasonal epidemics and periodic pandemics that are associated with significant morbidity, mortality^1^, and economic burden^2^. To date, vaccines have been the most effective measure to prevent and reduce the severity of IAV infection, and have substantially lowered the economic burden of IAV^3^. While these vaccination efforts have led to reduced IAV-associated morbidity and mortality overall, immunity and protection provided by current IAV vaccines is largely derived from systemic immunity in the form of anti-hemagglutinin (HA)-specific antibody responses that are predominantly strain matched^4,5^. Moreover, current IAV vaccines generate little to no de novo T cell responses within the lower-lung mucosa^6,7^. This local, lung-tissue resident immunity is poised for rapid responses to infection and is known to be critical for heterosubtypic protection^8–10^. Thus, while effective, the current vaccines do not generate multi-faceted immunity and broad protection against IAV, resulting in suboptimal protection against heterosubtypic, cross-group IAV infection^11,12^.

To combat the diverse repertoire of potential IAVs, a comprehensive IAV vaccine that drives both humoral and cellular immunity, and thus has the ability to overcome the genetic drift/shift of IAV, and could afford long-lasting, supra-seasonal protection against multiple IAV strains would be preferred^13,14^. Further, the current IAV vaccine production pipeline has an extended time lapse (6–9 months) in between when the vaccine strain for a given season is chosen and when the vaccine is available^15^. Lessons from the COVID-19 pandemic have reinforced the issues that may arise during an IAV pandemic which include vaccine production time and storage, vaccine protection against variant strains, and the importance of vaccine-induced mucosal immunity. Efforts to develop broadly protective IAV vaccines have utilized various platforms that include but are not limited to recombinant protein-based^16^, virus-like particle^17^, mRNA^18,19^, and polymeric nanovaccines^19–21^. Our own laboratory has previously reported on a novel, intranasally administered polyanhydride [copolymers of 1,8-bis(*p*-carboxyphenoxy)-3,6-dioxoctane (CPTEG) and 1,6-bis(*p*-carboxyphenoxy)hexane (CPH)] nanovaccine platform, known as IAV-nanovax, that generates systemic and mucosal T and B cell immunity and cross-strain protection against IAVs^22–24^. Moreover, this nanovaccine platform can be quickly produced and stored at ambient temperatures for prolonged periods of time, mitigating logistical problems that arise with current IAV vaccines^22,25^.

Our previous report demonstrated the efficacy of 20:80 CPTEG:CPH particles encapsulating IAV antigens hemagglutinin (HA) and nucleoprotein (NP) from an H1N1 strain (A/Puerto Rico/8/1934) and adjuvanted with CpG^24^. However, the immunity generated by nanovaccines encapsulating other IAV proteins and proteins from other IAV strains (e.g., H3N2) has not yet been determined. Interestingly, studies have suggested that targeting IAV proteins in addition to HA such as neuraminidase (NA), NP, and matrix protein (M1) may improve upon current vaccines^26–29^. Therefore, to evaluate the breadth and applicability of our polyanhydride nanovaccine platform, we investigated how several H3N2-nanovaccines incorporating combinations of IAV H3N2 proteins (i.e., HA, NA, NP, and M1) contribute to the generation of IAV-specific immunity and protection.

In this work, we demonstrate the efficacy of an intranasally (i.n.) administered, 20:80 CPTEG:CPH nanovaccine encapsulating CpG 1668 adjuvant and three combinations of proteins from IAV H3N2 (A/Hong Kong/1/1968) and (A/Aichi/2/1968): H3-nanovax (HA/NP), which is analogous to the IAV protein content of H1-nanovax (HA/NP), H3-nanovax (NA/NP), and H3-nanovax (NA/M1). Our results show that i.n. administration of these H3-nanovax formulations generated immunity in the form of systemic and mucosal B cell responses that were associated with IAV-specific IgG and IgA. Further, these vaccines generated both systemic and lung-tissue resident effector and memory CD4 and CD8 T cell responses and induced protection against homologous and heterosubtypic IAV challenges. Altogether, our results demonstrate that the polyanhydride nanovaccine platform possesses the capacity to “plug-and-play” new protein payloads from different strains of IAV to generate systemic and mucosal immunity as well as provide cross-strain protection against homologous and heterosubtypic IAV infection.

## Results

### IAV-nanovax particle characterization

**Fig. 1:**
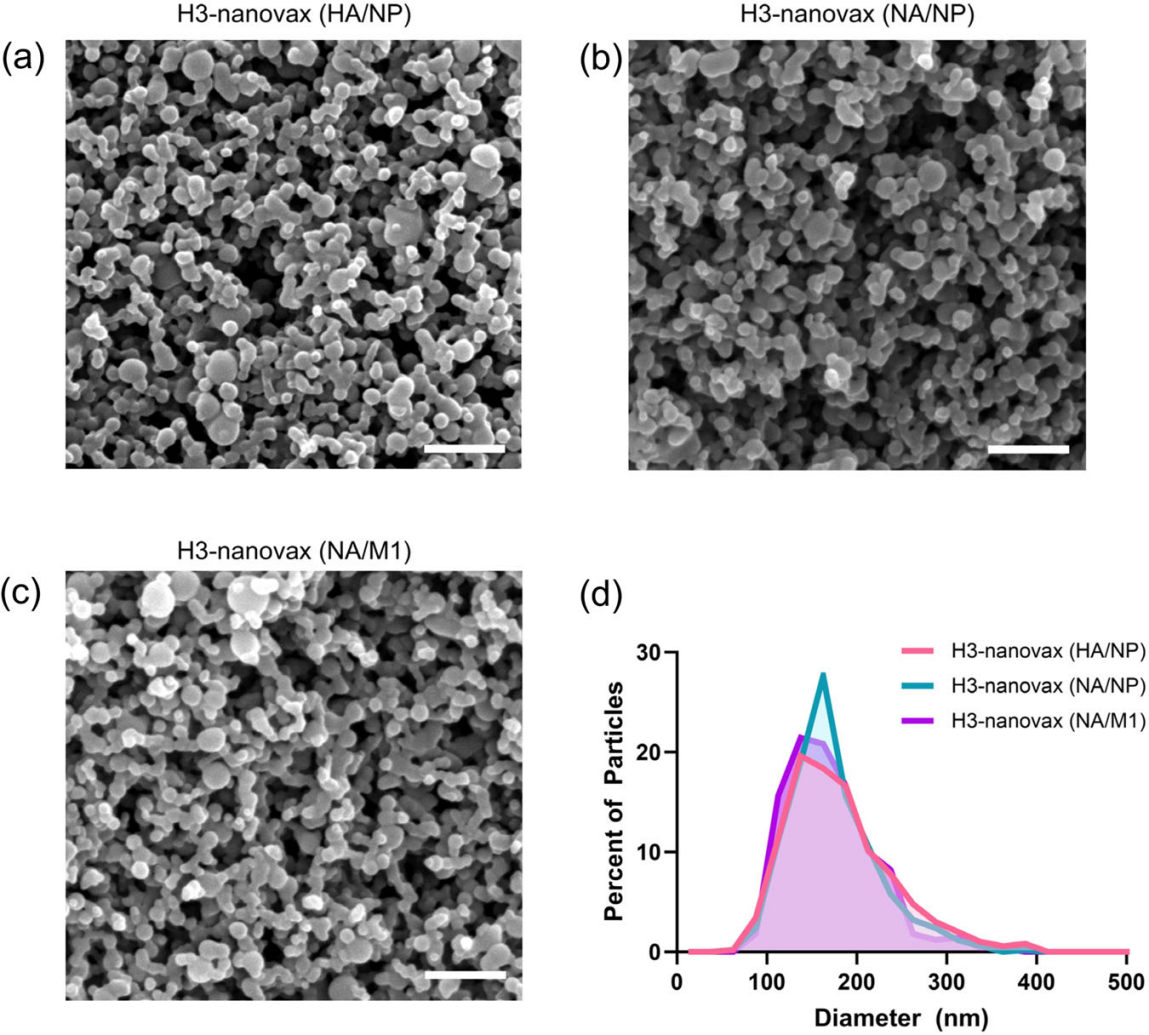
H3-nanovax particle characterization. Representative scanning electron micrographs of (**a**) H3-nanovax (HA/NP), (**b**) H3-nanovax (NA/NP), and (**c**) H3-nanovax (NA/M1). Scale bar represents 1 μm. **d** Size distribution of average particle diameter analyzing ~500 particles per image using ImageJ software.

Variations in the physicochemical properties of nanoparticles, such as size, shape, and interactions with their antigen payload can directly impact vaccine efficacy^30,31^. As such, we first characterized the morphology and size distribution of our H3-nanovax formulations. All three H3-nanovax formulations were found to be relatively spherical in morphology and partially aggregated, consistent with previous work^32^ (Fig. 1a–c). Analysis of representative scanning electron micrographs indicated that all three formulations had similar size distributions, with average diameters of 181 ± 61 nm (HA/NP), 173 ± 49 nm (NA/NP), and 171 ± 52 nm (NA/M1) (Fig. 1d). Additionally, the antigen encapsulation efficiency of each H3-nanovax formulation was determined by incubating the particles in 40 mM sodium hydroxide to rapidly degrade the polymer and release the encapsulated proteins. The released proteins were then quantified via a micro bicinchoninic acid assay. The antigen encapsulation efficiency of each H3-nanovax formulation was found to be 27 ± 2% (HA/NP), 24 ± 4% (NA/NP), and 9 ± 1% (NA/M1).

Additionally, we examined the integrity of the primary structure of the encapsulated proteins following their release from the nanoparticles relative to their original input unencapsulated counterparts. Similar to the input IAV proteins, the IAV proteins released from the nanoparticles were recognized by the appropriate antibodies specific for that IAV protein, and were observed to have a size consistent with their predicted molecular weights (HA: 71.3 kDa, NA: 76.8 kDa, NP: 54.4 kDa, and M1: 27.9 kDa) (Supplementary Fig. 1a–d). These results are consistent previous observations for proteins released from polyanhydride nanoparticles^22,33^ and suggests that the IAV proteins encapsulated in H3-nanovax formulations maintain their structural integrity as antigens.

### H3-nanovax formulations encapsulating IAV proteins induce lung and lymph node resident GC B cells and viral protein targeted antibody

**Fig. 2:**
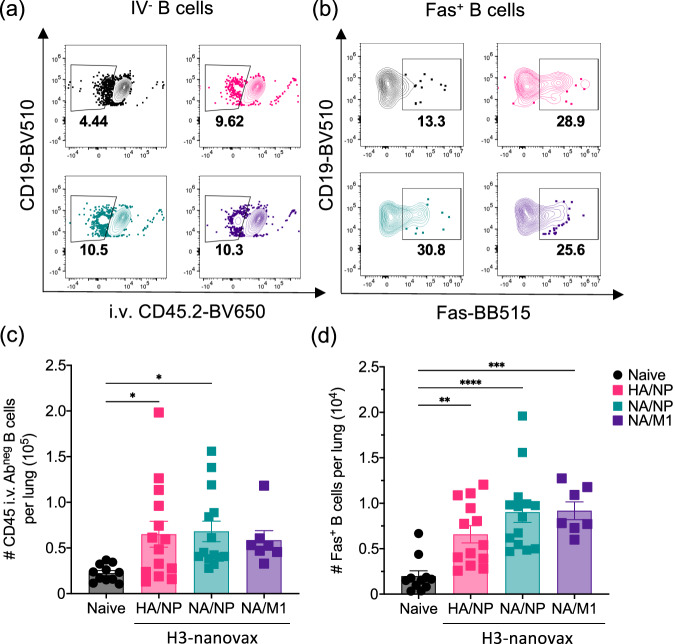
H3-nanovax encapsulating IAV proteins induce lung-resident B cell responses. C57BL/6 mice were vaccinated i.n. with 500 μg of H3-nanovax encapsulating CpG + either HA/NP (Pink), NA/NP (Blue), NA/M1 (Purple), or left unvaccinated (naïve). Representative flow plots of total CD45i.v.Ab^-^CD19^+^ B cells (**a**) and Fas^+^ (CD45i.vAb^-^CD19^+^Fas^+^) B cells (**b**) within the lungs. At 45 days post-vaccination, lung-resident (i.e., CD45i.v.Ab^-^) B cells (**c**) and Fas^+^ B cells (**d**) were enumerated. Error bars, mean ± s.e.m. Data representative of two (**c**, **d**; M1/NA: *n* = 3–4 mice/group) or three (**c**, **d**); Naïve, HA/NP, and (NA/NP: *n* = 4–5 mice/group) independent experiments. *P < 0.05, **P < 0.01, ***P < 0.001, ****P < 0.0001 (One-way ANOVA with Tukey’s multiple comparisons test).

B cell responses, particularly in the form of class-switched germinal center (GC) B cells producing high-affinity antibodies targeted against IAV proteins, have been shown to be crucial for optimal control and protection against subsequent IAV infection^34,35^. Therefore, we first analyzed total B cell responses following i.n. vaccination wherein mice received a prime + boost (spaced 14 days apart) regimen of 500 µg H3-nanovax encapsulating one of three protein payload combinations (e.g. HA/NP, NA/M1, or NA/NP) + CpG or received no vaccine (naïve). At 45 or 50 days after the initial vaccination, mice were administered fluorophore conjugated anti-CD45.2 mAb intravascularly (i.v.) 3 min prior to euthanasia to allow for discrimination between circulating (i.e., CD45ivAb^+^) and tissue protected (i.e., CD45ivAb^-^) cell populations^36^ (see Supplementary Fig. 2). Following harvest of lung and lung-draining lymph nodes (dLN), we observed an increase in total B cells present in the lungs for all three H3 based nanovaccines relative to naïve mice (Fig. 2a, c). Consistent with this increase in total B cells following vaccination, lung-tissue resident Fas^+^ B cells (CD45i.vAb^-^CD19^+^Fas^+^) were also increased for all three vaccine groups (Fig. 2b, d). As lung-tissue resident B cells have been associated with both germinal center (GC) B cells and lung tissue-resident B cells (B_RM_) we next examined whether the H3-nanovax formulations induced GC and B_RM_ cells. Germinal center B cells (CD45i.vAb^-^CD19^+^Fas^+^GL-7^+^) (Supplementary Fig. 3a, c) from vaccinated mice were increased in both the lungs (Supplementary Fig. 3a, c) and lung-draining dLN (Supplementary Fig. 4: CD45i.vAb^-^CD19^+^Fas^+^GL7^+^) relative to their naïve counterparts for the H3-nanovax HA/NP and NA/NP groups. In contrast germinal center B cells were increased for H3-nanovax NA/M1 only in the dLN. As recent reports have detailed the importance of lung tissue-resident memory B cells (B_RM_) in contributing to early plasmablast responses as well as protection during subsequent IAV infections^37,38^, we next examined if H3-nanovax induced B_RM_. Forty-five days post vaccination, we observed an increase in B_RM_ (CD45i.vAb^-^CD19^+^ Fas^+^GL7^-^CD38^+^CD69^+^IgM^-^CXCR3^+^) (Supplementary Fig. 3b, d) in the lungs for the HA/NP and NA/NP H3-nanovax formulations which mirrored the increase seen in GC B cells (Supplementary Fig. 3a, c). Consistent with our previously reported findings^24,39^, the presence of the IAV protein payload was necessary to increase GC and B_RM_ B cell populations after H3-nanovax vaccination, as we did not observe significant increases in these populations when a control CpG-only nanovax (i.e. no IAV protein payload) was used (Supplementary Fig. 5a, b).

Consistent with these findings, while all three H3-nanovax vaccinated groups generated total H3N2 IAV virion-specific IgG (Supplementary Fig. 6a, b), total H3N2 IAV virion-specific serum and BAL IgG were significantly increased versus the other vaccine groups in mice that received H3-nanovax (NA/NP), whereas H3-nanovax (HA/NP) was elevated relative to naïve mice in both serum and BAL, and H3-nanovax (NA/M1) only showing increases relative to naïve mice in serum IgG (Supplementary Fig. 6a, b). Further this result is consistent with the reduced lung GC response observed after vaccination with H3-nanovax (NA/M1) (Supplementary Fig. 3a, c). As IgA production is also important for protection against IAV^40,41^ we next quantified total H3N2 IAV virion-specific IgA levels in the BAL^42^. The total IAV-specific IgA in the BAL demonstrated a pattern similar to serum IgG. H3-nanovax (NA/NP) induced a significant increase in IgA when compared to naïve mice. The H3-nanovax (HA/NP) and (NA/M1) vaccines also showed higher responses than naïve mice, albeit not significantly (Supplementary Fig. 6c).

**Fig. 3:**
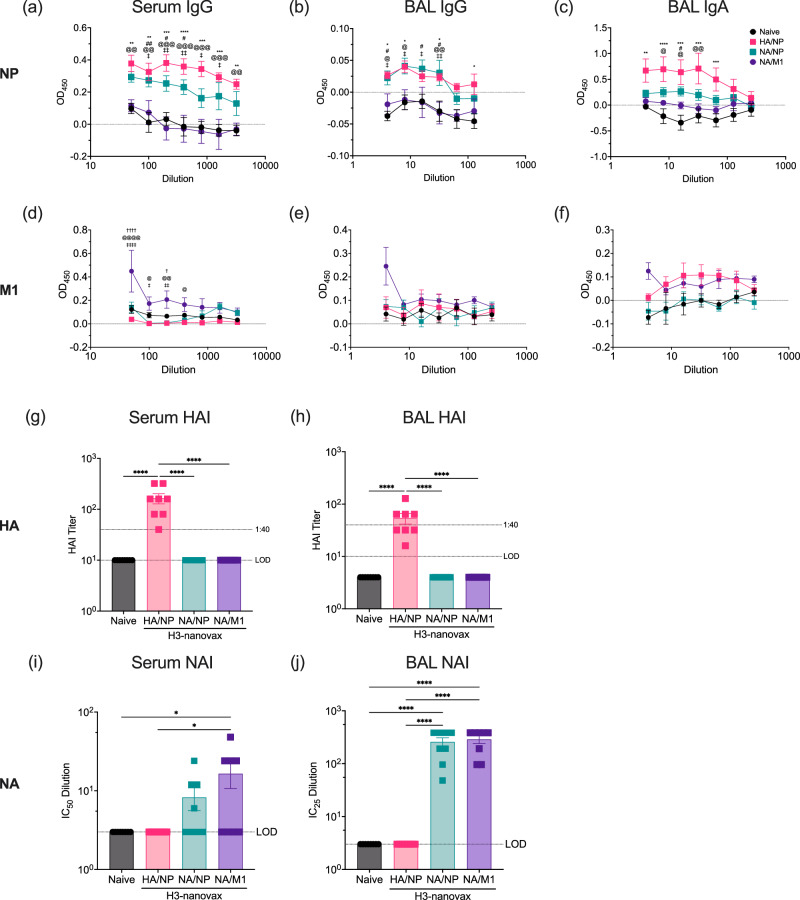
H3-nanovax encapsulating IAV proteins induces payload targeted systemic and lung-local IAV-specific antibody responses. C57BL/6 mice were vaccinated with H3-nanovax encapsulating CpG + either HA/NP (Pink), NA/NP (Blue), NA/M1 (Purple), or left unvaccinated (naïve). At 45 days post-vaccination, serum and BAL were collected and serum and BAL IgG (**a**, **b**, **d**, **e**) and BAL IgA (**c**, **f**) specific for NP (A/Hong Kong/1/68, H3N2) and M1 (A/Aichi/2/68, H3N2) were quantified via ELISA. As a measure of anti-HA specific antibody against IAV A/Hong Kong/1/68 (H3N2), HAI titers in the serum and BAL were quantified (**g**, **h**). Shown is the 1:40 titer (dotted line) that is thought to be the threshold for a protective titer. LOD (dotted line) = Limit of Detection. Anti-NA antibody in serum and BAL (**i**, **j**) were quantified via fluorescence-based NAI. LOD (dotted line) = Limit of Detection; Error bars, mean ± s.e.m. Data representative of two independent experiments with *n* = 4 mice/group. **a**–**j** HA/NP vs. naïve: *P < 0.05, **P < 0.01, ***P < 0.001, ****P < 0.0001; NA/NP vs. naïve: #*P* < 0.05, ##*P* < 0.01, ###*P* < 0.001, ####*P* < 0.0001; NA/M1 vs. naive: †*P* < 0.05, ††*P* < 0.01, †††*P* < 0.001, ††††*P* < 0.0001; HA/NP vs. NA/M1: @*P* < 0.05, @@*P* < 0.01, @@@*P* < 0.001, @@@@*P* < 0.0001, NA/NP vs. NA/M1: *ǂP* < 0.05, *ǂǂP* < 0.01, *ǂǂǂP* < 0.001, *ǂǂǂǂP* < 0.0001, (Two-way ANOVA with Tukey’s multiple comparison test). (**g**, **h**) ****P < 0.0001 (One-way ANOVA with Tukey’s multiple comparisons test).

These variations in total IAV-specific antibody production may be linked to responses directed towards the individual viral proteins within each vaccine formulation as well as related to the proteins’ relative abundance and/or exposure on virus particles (intact or broken) used as the target for the assay and thus could underrepresent the level of anti-IAV specific antibody present. Therefore, we next measured antibody responses using targeted assays against the individual viral proteins (HA, NA, NP, and M1) encapsulated within the nanoparticles as studies have suggested antibody against HA, NA, NP, and M1 can confer protection^26,42–44^. Therefore, to first determine the level of anti-NP antibody induced, we quantified NP-specific IgG and IgA antibody via ELISA utilizing NP from A/Hong Kong/1/68 (H3N2) (Fig. 3a–c) as the antigen target. As expected, the serum and BAL samples from naïve mice and those administered with H3-nanovax (NA/M1) did not exhibit elevated levels of anti-NP antibody. However, significant increases in anti-NP IgG were observed in the serum (Fig. 3a) for both H3-nanovax formulations that encapsulated NP (H3-nanovax (HA/NP) and H3-nanovax (NA/NP)). Similarly, when we measured anti-NP IgG in BAL samples, significant increases were observed for both NP encapsulating vaccines (Fig. 3b; H3-nanovax (HA/NP) and H3-nanovax (NA/NP)). Examination of lung-local IgA production against NP (Fig. 3c) also showed increased NP-specific IgA for those H3-nanovax formulations that encapsulated NP (H3-nanovax (HA/NP) and H3-nanovax (NA/NP)), but only H3-nanovax (HA/NP) was significantly increased. No reactivity was observed when a His-tagged SARS-CoV-2 S1 subunit protein was used as a target for the ELISA (Supplementary Fig. 7) demonstrating that the reactivity observed in Fig. 3a–c is to IAV-NP rather than His-tag specific. Next, we examined the ability of H3-nanovax (NA/M1) to generate anti-M1 antibodies relative to the formulations not encapsulating M1. As with NP, we quantified serum anti-M1 IgG and BAL anti-M1 IgG and IgA. While H3-nanovax (NA/M1) demonstrated significant increases in serum IgG relative to non-M1 encapsulating formulations (Fig. 3d), we did not observe significant increases in anti-M1 IgG or IgA in the BAL (Fig. 3e, f), a result consistent with the reduced lung GC response observed after vaccination with H3-nanovax (NA/M1) (Supplementary Fig. 3a, c).

We next measured antibody responses targeted against the surface proteins of IAV. First, we quantified systemic and mucosal anti-HA antibody by performing hemagglutinin inhibition^34^ assays on serum and BAL from naïve and vaccinated mice. As expected, H3-nanovax encapsulating HA demonstrated an HAI titer exceeding the protective threshold of 1:40^45^ while naïve and mice vaccinated with H3-nanovax formulations lacking HA (i.e., NA/M1 and NA/NP) did not produce any anti-HA antibody above the limit of detection (LOD) (Fig. 3g, h). When we next quantified serum and BAL anti-NA antibody via neuraminidase inhibition^46^ assays expressed as IC_50_ and IC_25_ titers for serum and BAL respectively, we observed that samples from mice administered H3-nanovax formulations encapsulating NA (i.e., NA/M1 and NA/NP) inhibited NA-activity at levels well above the limit of detection (LOD) while samples from naïve mice and mice receiving H3-nanovax lacking NA (i.e., HA/NP) displayed no significant antibody mediated NA-inhibition above the LOD (Fig. 3i, j). Although anti-NA activity with H3-nanovax formulations encapsulating NA (i.e., NA/M1 and NA/NP) exhibited ~40–45% inhibition of NA activity for BAL samples it did not reach 50% therefore the data is displayed as an IC_25_ to reflect the activity observed.

Interestingly, the observation that H3-nanovax (NA/M1) formulation, despite similar levels of anti-NA antibody in the BAL, had reduced GC B cell and total BAL antibody-based immunity (Supplementary Fig. 3) when compared to the H3-nanovax (NA/NP) formulation could suggest that responses to the individual protein payloads within the nanovaccine may be independent and/or in this case could relate to the reduced encapsulation efficiency of the H3-nanovax (NA/M1) formulation. However, altogether these results suggest that all formulations of H3-nanovax induce strong B cell responses in the lungs (Fig. 2, Supplementary Fig. 3) and dLN (Supplementary Fig. 4) and produce antibodies specific and selective to the IAV proteins individually encapsulated within the nanoparticles (Fig. 3). Furthermore, the data presented in Supplementary Fig. 6 (intact virions) and Fig. 3 are consistent with the finding that the protein payloads released from the nanoparticle are structurally intact, immunogenic and induce antibody responses against the corresponding IAV proteins produced during IAV infections.

### H3-nanovax formulations promote lung and lymph-node resident T cell responses

**Fig. 4:**
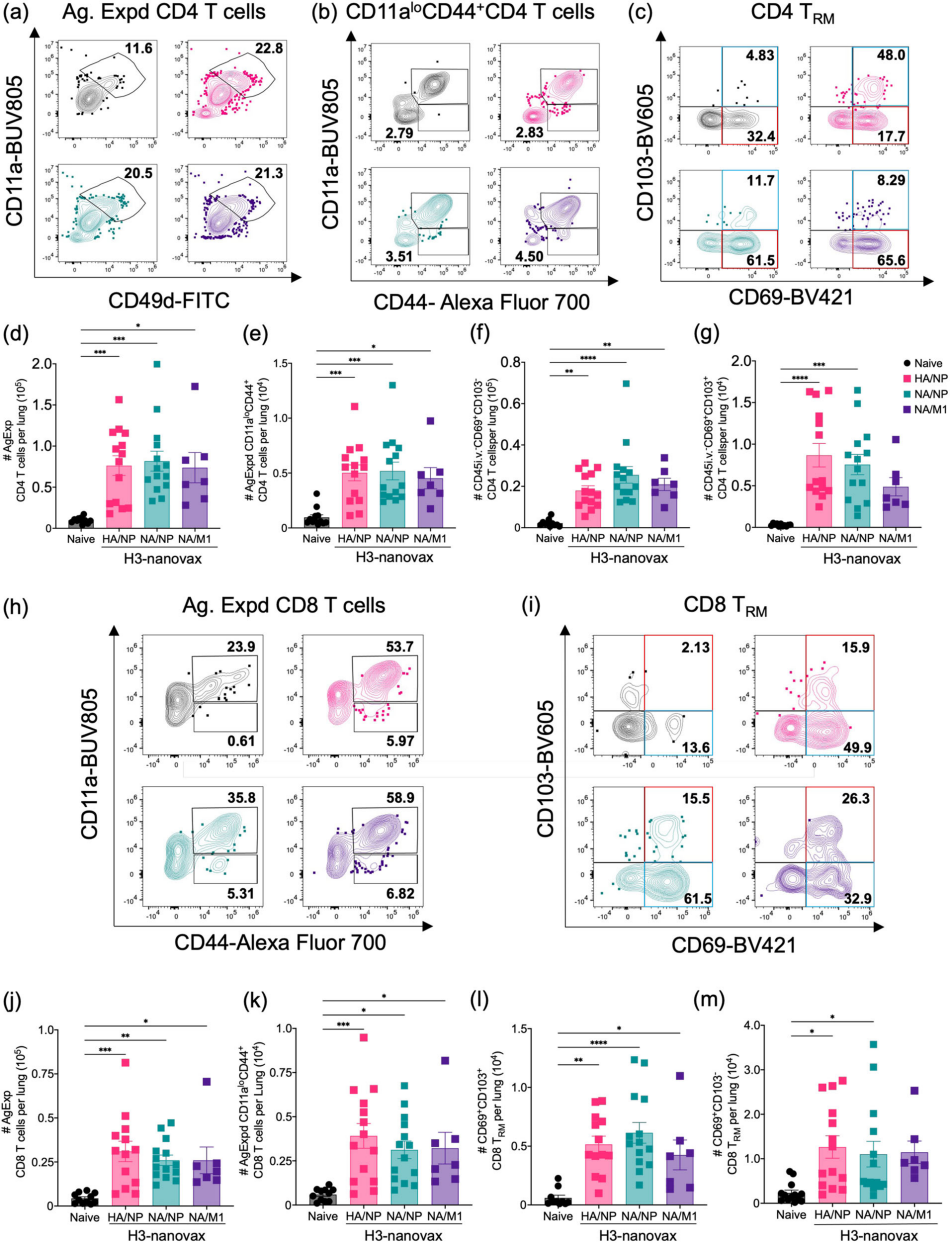
Vaccination with H3-nanovax encapsulating IAV proteins induces robust lung-resident T cell responses. C57BL/6 mice were vaccinated with H3-nanovax encapsulating CpG + either HA/NP (Pink), NA/NP (Blue), NA/M1 (Purple), or left unvaccinated (naïve). Representative flow plots and enumeration of lung and airway resident antigen-experienced CD4 (**a**–**g**) and CD8 T cells (**h**–**m**). At 45 days post-vaccination, the total number of lung (CD11a^hi^CD44^+^) (**d**, **j**) and airway (CD11a^lo^CD44^+^) (**e**, **k**) resident antigen-experienced CD4 (CD45i.vAb^-^CD4^+^CD49d^+^) and CD8 (CD45i.vAb^-^CD8α^+^CD44^+^) T cells were enumerated. T_RM_ within the lung resident, antigen-experienced CD4 and CD8 T cells were further characterized based on expression of CD69 and CD103(CD69^-^CD103^+^ CD4): (**c**) (red box), (**f**); CD69^+^CD103^+^ CD4: (**c**) (blue box), (**g**); CD69^+^CD103^+^ CD8: (**i**) (red box), (**l**); CD69^+^CD103^-^ CD8: (**i**) (blue box), **m** Error bars, mean ± s.e.m. Data representative of two (**a**–**m**; M1/NA: *n* = 3–4 mice/group) or three (**a**–**m**; Naïve, HA/NP, and NA/NP: *n* = 4–5 mice/group) independent experiments. *P < 0.05, **P < 0.01, ***P < 0.001, ****P < 0.0001 (One-way ANOVA with Tukey’s multiple comparisons test).

CD4 T cell help is important for the production of high-affinity class-switched antibodies generated in GCs as well as modulating the immune response to promote effective clearance of IAV^42,47^. CD8 T cells contribute to protection against IAV by eliminating infected cells via their cytotoxic capabilities, and CD8 T cells in the form of lung tissue-resident memory (T_RM_) are especially important to protection as they are poised for rapid responses against subsequent IAV exposures via their positioning, sensing, and alarm functions^48,49^. Together these CD4 and CD8 T cells in the lung mucosa are thought to be important for broad-based protection against heterosubtypic IAVs and the induction of these responses serve as an important component of our mucosal vaccine design^8,48,50,51^. To evaluate the capacity of H3-nanovax formulations to induce vaccine-specific CD4 and CD8 T cell responses in the lungs and lung draining lymph nodes, mice were administered a prime + boost vaccination as described above, and tissues were collected 45 or 50 days after the primary immunization. To measure this T cell response, we initially made use of a well validated surrogate marker-based approach that has proven useful in mice and humans that is based upon expression levels of CD8a and CD11a, or CD49d and CD11a, to track antigen-experienced (Ag. Expd) CD8 and CD4 T cells, respectively^24,52–56^. This technique offers several advantages in that it provides a snapshot of the full T cell response against the pathogen/immunogen and is not biased by only examining the immunodominant or predicted epitopes from individual proteins from a specific virus strain, and does not require prior knowledge of all T cell epitopes or epitope density for a given pathogen. We observed that all three nanovaccine formulations generated significant numbers of antigen-experienced CD4 T cells (Ag. Expd CD4; CD4^+^CD11a^hi^CD49d^+^) in the lungs relative to naïve mice (Fig. 4a, d). These antigen-experienced CD4 T cells expressed markers used to classically define canonical lung T_RM_ (CD69^+^CD103^-^, red box)^51,57^ (Fig. 4c, f, red box) as well as an additional lung-tissue resident population of CD4 memory T cells (CD69^+^CD103^+^, Fig. 4c, blue box), a subset which has been suggested to possess cytotoxic capabilities in other studies (Fig. 4c, g)^47,58^. Both subsets aside from CD69^+^CD103^+^ cells CD4 memory T cells in the H3-nanovax (NA/M1) were significantly increased in mice vaccinated with H3-nanovax formulations relative to naïve counterparts. Similarly, the antigen-experienced (Ag. Expd CD8; CD11a^hi^CD44^+^) CD8 T cell response against all three H3-nanovax formulations was also increased in the lungs compared to naïve mice (Fig. 4h, j). This increase was observed in two populations of tissue-resident cells expressing markers associated with tissue resident memory. The first CD8 T_RM_ (CD69^+^CD103^+^) (Fig. 4i, l, red box) exhibited a uniform lung T_RM_ signature classically associated with T_RM_ [low Eomesodermin (Eomes), high CD49a, high CXCR3, and low CX3CR1] (Supplementary Fig. 8). The other (i.e. CD69^+^CD103^-^ cells) (Fig. 4i, m, blue box) expressed some of these markers and may contain some CD8 T_RM_, however this population was more heterogenous in Eomes, CD49a, CXCR3, and CX3CR1 expression compared to their CD69^+^CD103^+^ counterparts, suggesting it could be a transitional population (Supplementary Fig. 8)^50,59,60^. Similar to our B cell findings and consistent with our prior reports^24,39^, the presence of the IAV protein payload was necessary to significantly increase these CD4 and CD8 T cell populations after H3-nanovax vaccination, as we did not observe significant increases in these populations when a control CpG-only nanovax (i.e., no IAV protein payload) was used (Supplementary Fig. 5c–h).

In addition to T cells in the lung parenchyma, airway-resident T cells contribute to protection against IAV via airway surveillance and cytokine production^51,61,62^. As past studies have shown that T cells downregulate CD11a following entry into the airways, we therefore utilized the change in CD11a expression to enumerate CD11a^lo^CD44^+^ T cells that may be airway-resident T cells^61,63^ (CD45i.vAb^-^CD11a^lo^CD44^+^ T cells: CD4^+^ or CD8^+^). Similar to T cells in the parenchyma, we observed significant increases for both antigen experienced CD11a^lo^CD44^+^ CD4 (Fig. 4b, e) and CD8 (h, k) T cells following all vaccination regimens. While the CD11a^lo^CD44^+^ methodology may not capture all airway-resident cells as newly arriving T cell may not have downregulated CD11a yet, altogether our results suggest that all three H3-nanovax formulations induce both airway and lung interstitial CD4 and CD8 T cells. Analogous increases in antigen-experienced CD4 and CD8 T cell responses were also observed in the dLN of vaccinated mice, demonstrating the vaccine’s ability to drive systemic T cell responses (Supplementary Fig. 9).

To further quantify the T cell response at a specific defined epitope level, we next utilized tetramers specific for H3N2 defined NP_366-374_ and M1_128-135_ epitopes in C57BL/6 mice. Our results demonstrate that the H3-nanaovax formulations containing NP (e.g., HA/NP and NA/NP) induces CD8 T cells against NP_366_ whereas those lacking NP (e.g., NA/M1 and CpG only) do not (Supplementary Fig. 10a–c). Similarly, only the formulation containing M1 induced CD8 T cells against the M1_128_ epitope (Supplementary Fig. 10d, e). Interestingly, despite inducing similar levels of total Ag. Expd CD8 T cells (Fig. 4) we observed that there were reduced numbers of both antigen-experienced and lung-resident memory T_RM_ CD8 T cells specific for NP_366_ in the lungs of mice vaccinated with H3-nanovax (HA/NP) relative to H3-nanovax (NA/NP) (Supplementary Fig. 10a, b). This difference was not observed in the draining lymph nodes (Supplementary Fig. 10c). Altogether, these results demonstrate that all three of the H3-nanovax formulations are capable of inducing antigen-experienced memory CD4 and CD8 T cell responses in the lungs, airways, and dLN as well as drive the induction of T_RM_ in the lungs i.e., cell populations which are known to be important for protection against heterosubtypic IAV infection^8,43,48^, but that for T cells specific for any single virus epitope there could be, depending on the vaccine formulation utilized, some differential accumulation/localization of the T cells.

### H3-nanovax formulations provide protection against homologous and heterosubtypic IAV infections

**Fig. 5:**
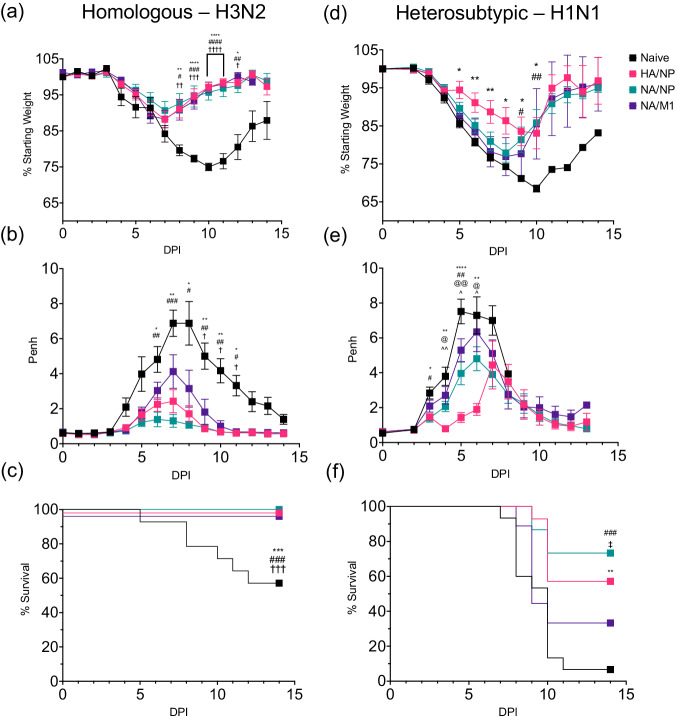
H3-nanovax formulations provide protection against homologous and heterologous IAV infection. C57BL/6 mice were vaccinated with H3-nanovax encapsulating CpG + either HA/NP (Pink), NA/NP (Blue), NA/M1 (Purple), or left unvaccinated (naïve). At 45 days post vaccination, mice were challenged with a lethal dose of homologous (**a**–**c**) A/Hong Kong/1/1968 (H3N2) or heterologous (**d**–**f**) A/Puerto Rico/8/1934 (H1N1). Subsequently, morbidity (i.e., weight loss (**a**, **d**), lung-airway resistance (Penh) (**b**, **e**)) and mortality (**c**, **f**) were monitored daily. Error bars, mean ± s.e.m. Data representative of 3 independent experiments with *n* = 4–5 mice/group. HA/NP vs. naïve: *P < 0.05, **P < 0.01, ***P < 0.001, ****P < 0.0001; NA/NP vs. naïve: #*P* < 0.05, ##*P* < 0.01, ###*P* < 0.001, ####*P* < 0.0001; NA/M1 vs. naive: †*P* < 0.05, ††*P* < 0.01, †††*P* < 0.001, ††††*P* < 0.0001; HA/NP vs. NA/M1: @*P* < 0.05, @@*P* < 0.01, @@@*P* < 0.001, @@@@*P* < 0.0001; NA/NP vs. NA/M1: ǂ*P* < 0.05; HA/NP vs. NA/NP: ^*P* < 0.05, ^^*P* < 0.01 (**a**, **b**, **d**, **e**) (Two-way ANOVA with Holm-Sidak multiple comparison test). (**c**, **f**) (Mantel-Cox Log rank test).

**Fig. 6:**
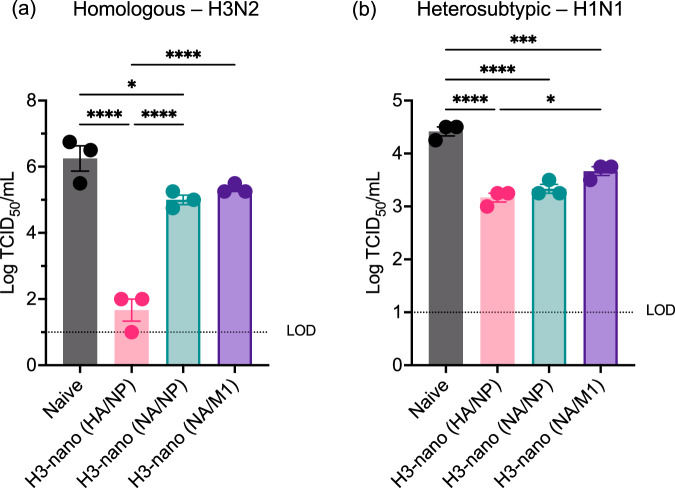
H3-nanovax formulations reduce viral load against homologous and heterosubtypic infection. C57BL/6 mice were vaccinated (H3-nanovax (HA/NP; Pink), (NA/NP; Blue), (NA/M1; Purple), or left unvaccinated (naïve)) as described in Fig. 2. 32 days following prime vaccination, mice were infected with 390 TCIU IAV A/Hong Kong/1/68 (**a**) or 1108 TCIU A/Puerto Rico/8/34 (**b**). At 3 days post infection, lungs were harvested and mechanically homogenized. TCID_50_/mL was measured in triplicate for each mouse. Error bars, mean ± s.e.m. LOD (dotted line) = Limit of Detection. Data representative of one (*n* = 3 mice/group) independent experiment *P < 0.05, ***P < 0.001, ****P < 0.0001, (One-way ANOVA with Tukey’s multiple comparisons test on log transformed data).

Our previous results demonstrated that H3-nanovax HA/NP, NA/M1, or NA/NP are all capable of generating systemic and local (i.e., lungs/airways) immunity following vaccination compared to naïve counterparts (Figs. 2–4). This includes the expansion of GC B cells, generation of IAV antigen-specific IgG and IgA, and the induction of antigen-experienced and lung- and airway-resident T cells and T_RM_. Not surprisingly, the focus of this immunity was targeted to the antigens found within the H3-nanovax formulations (Fig. 3, Supplementary Fig. 10). Therefore, we next determined whether such immunity translated to reduction in IAV-associated morbidity and mortality following lethal dose homologous and heterosubtypic IAV challenge. To this end, 45 days following the initial vaccination with our three H3-nanovax formulations, naïve and vaccinated mice were challenged with a lethal dose of homologous IAV (A/Hong Kong/1/68, H3N2) and mortality and morbidity (i.e., weight loss, lung-airway resistance (Penh)) were monitored daily. Following homologous IAV challenge, naïve and vaccinated mice from all three H3-nanovax groups had similar morbidity as demonstrated by limited weight loss and airway resistance at early timepoints (Fig. 5a, b). However, by 7 days post-challenge, naïve mice demonstrated increased morbidity in contrast to vaccinated mice, who began to regain body weight and showed significantly less respiratory distress. Further, while >40% of naïve mice succumbed to this lethal dose challenge, all three groups of vaccinated mice recovered and demonstrated 100% survival against homologous IAV challenge (Fig. 5c). While antibody can be sufficient to mediate protection against homologous IAV, our nanovaccines also generated T cell responses (Fig. 4, Supplementary Figs. 9–10). Since T cells are known to help confer protection against heterosubtypic IAV, we therefore examined whether our three H3-nanovax formulations could confer protection against a highly stringent, lethal dose heterosubtypic H1N1 IAV (A/Puerto Rico/8/34) challenge. Importantly, all three H3-nanovax formulations demonstrated protection. The H3-nanovax (HA/NP) and (NA/NP) groups showed the greatest level of protection post-heterosubtypic challenge (Fig. 5f) with the H3-nanovax (NA/M1) mice conferring protection albeit to a lesser extent compared to the other vaccinated groups. Similar protection was observed when examining morbidity. While mice vaccinated with H3-nanovax (HA/NP) exhibited the most pronounced reduction in morbidity (days 6–9 weight loss, days 4–6 lung function) compared to the other groups (Fig. 5d, e), both the H3-nanovax (NA/NP) and (NA/M1) mice also showed reduced weight loss (NA/NP days 9–10); NA/M1 days 9–10 and improved lung function (NA/NP days 4–6; NA/M1 days 4–6) vs. naïve mice. To examine whether this reduction in morbidity and mortality and associated immunity corresponded with a reduced viral load in the lungs, we next quantified viral titers post challenge with homologous or heterosubtypic IAV. To this end, mice were vaccinated with the H3-nanovax formulations or left naïve as a control and on day 32 post vaccination challenged with either a 390 TCIU of A/Hong Kong/1/68 (H3N2, homologous) or 1108 TCIU A/Puerto Rico/8/34 (H1N1, heterosubtypic) dose of IAV. Three days following IAV challenge, lungs were homogenized, and viral titers were assessed via TCID_50_. Consistent with our observed HAI results (Fig. 3) and the known role of antibody to HA in reducing viral infection and protecting against matched virus strains by providing sterilizing immunity in some cases by blocking HA binding to sialic acids and entry of the virus into cells, H3-nanovax (HA/NP) vaccinated mice challenged with homologous H3N2 challenge displayed reduced viral titers relative to naive mice (Fig. 6a). Similarly, mice vaccinated with the NA/NP and NA/M1 H3-nanovax formulations demonstrated a substantial reduction (greater than a 1 log reduction) in virus, a result that would be consistent with the observed antibody, B cell and T cell immune responses (Figs. 2–4, Supplementary Figs. 3, 4, 6, 9, 10) and the known role for non-neutralizing Ab (e.g., Ab targeting NA, NP, M1) (Figs. 2, 3, Supplementary Figs. 3, 4, 6) and CD4 and CD8 T cells (Fig. 4, Supplementary Figs. 9, 10) in providing protection after the virus enters the first cell (Fig. 5)^43,64^. Importantly, we observed a similar significant reduction in pulmonary virus titers in the lungs of mice from all three H3-nanovax groups that were challenged with heterosubtypic viruses, a result consistent with the observed immunity generated by the three H3-nanovax formulations and the known role for non-neutralizing Ab, CD4 and CD8 T cells, and local tissue-resident immunity in protection against heterosubtypic IAV infections. Together, these data suggest that all three H3-nanovax formulations generate systemic and mucosal immunity that is associated with reduced viral titers and protection against lethal homologous and heterosubtypic IAV challenge.

## Discussion

This study complements other research performed by ourselves and colleagues demonstrating the flexibility of this nanovaccine platform in its ability to encapsulate multiple antigens against various respiratory pathogens^39,65^. Herein, we have demonstrated the “plug-and-play” capability of the polyanhydride nanovaccine platform by developing H3-nanovax formulations containing various combinations of H3N2 IAV antigens (HA, NA, NP, and M1). These formulations were capable of inducing systemic and mucosal B and T cell immunity as well as protect against both homologous and heterosubtypic IAV infections, similar to previous reports on H1N1 IAV-nanovax and the same nanovaccine nanoparticle backbone utilized in the context of RSV^24,39^.

Regarding the H3-nanovax formulations tested herein, the specific proteins utilized were chosen based on their importance to infection/viral replication as well as the known contributions of protective immunity against them^26,27,43^. HA was chosen as it has traditionally been targeted by IAV vaccines due to its critical role in the initial cellular entry of the virus during infection and the fact that neutralizing antibodies against the variable region of HA can limit cellular entry of IAV and thus provide sterilizing immunity to matched IAV strains^18,66,67^. Additionally, it is the only IAV protein antigen whose content is regulated in the currently licensed IAV vaccines^68^. As studies have suggested that existing levels of anti-NA antibody may be a better predictor of protection in humans compared to anti-HA and may contribute to protection and lower disease severity by restricting the release of newly synthesized influenza virions from cells^69–71^, we also targeted NA for two of our H3-nanovax formulations (H3-nanovax NA/NP and H3-nanovax NA/M1). The internal IAV proteins NP and M1 that were encapsulated in our H3-nanovax formulations were chosen based on their increased conservation among IAV strains as well as their ability to induce T cell immunity and protective antibody responses. NP is abundantly expressed during infection and protective T cell epitopes are found in mice and humans, including an IAV NP epitope shown to induce CD8 T cells that can cross-react against IAV and IBV in HLA-B37 individuals^72^. Moreover, non-neutralizing anti-NP antibodies promote recovery and lower viral titers following IAV infection via antibody dependent cellular cytotoxicity (ADCC) as well as associate with viral proteins/virions to form immune complexes targeted by dendritic cells leading to increased antigen presentation and T cell immunity^43,64,73^. Reports suggest that antibody against M1 may also induce NK cell mediated ADCC^44^ and therein contribute to protection. Further, studies on the sequence of IAV M1 show this protein exhibits 95% amino acid sequence identity among strains circulating in humans and has also been identified as a conserved immunodominant CTL epitope in HLA-A2 and HLA-C*08 individuals^74–77^. Similarly, CD4 T cell epitopes in M1 and NP have been described for DRB1*0101 individuals^77^. Natural IAV infection induces B and T cell immunity against a number of viral proteins suggesting that the gold standard for broad-based vaccines that induce long-lived protection would be to mimic the diverse repertoire of immunity generated by natural infection without the associated morbidity and mortality, therein creating an environment that makes it harder for variant viruses to escape protection. Given that specific immunity targeted against each IAV antigen can have unique contributions to protection against IAV and our data demonstrating, as expected, that immunity generated by the H3-nanovax was specific to the included IAV protein payloads (Fig. 3, Supplementary Fig. 10), future iterations of IAV-nanovax containing multiple (3 + ) viral antigens from one or multiple virus strains (i.e., H1N1 and H3N2) could further broaden immunity leading to even greater protection against circulating seasonal, pandemic, and pre-pandemic IAV strains.

Among the H3-nanovax formulations tested, we observed that all three induced significant B cell responses in the lung/dLN (Fig. 2, Supplementary Figs. 3, 4) and found IAV-specific (i.e., virion targeted) systemic (IgG) as well as mucosal (IgG and IgA) antibodies (Fig. 3, Supplementary Fig. 6a–c). Further examination of the anti-IAV antibody response demonstrated that specificity of the antibodies generated matched the viral proteins within the respective formulation’s payload (Fig. 3). Indeed, antibody against each specific antigen was increased after IAV-nanovax vaccination to levels greater than observed in naïve mice; HAI titers were above the protective 1:40 threshold^45^ for H3-nanovax encapsulating HA (i.e., H3-nanovax HA/NP), anti-NA Ab levels were increased after vaccination with nanoparticles encapsulating NA (i.e., H3-nanovax NA/NP, H3-nanovax NA/M1) as shown via NAI, anti-M1 IgG was increased in the serum of H3-nanovax (NA/M1) mice, and systemic anti-NP IgG and lung-local anti-NP IgG and IgA were increased by NP encapsulating nanoparticles (i.e., H3-nanovax HA/NP, H3-nanovax NA/NP). It should be noted however that H3-nanovax (NA/NP) drove the highest total (i.e., total virion) IAV-specific IgG response (Supplementary Fig. 6a, b). Further, the induction of these protein specific responses together with our analysis of the protein payloads liberated from the nanovaccines suggest, as we have previously observed, that there is minimal impact on the integrity and antigenicity of the encapsulated proteins as they generate virus specific antibodies capable of recognizing intact IAV virions, the source IAV proteins^22,33^, and inhibiting the effector functions of HA and NA (Fig. 3g–j).

Herein we report the ability of the IAV nanovaccine platform to induce lung B_RM_ responses (Supplementary Fig. 3b, d), which studies have suggested are important for early plasmablast responses as well as protection against subsequent IAV challenge^37,38,78^. Importantly these B_RM_ cells are CXCR3^+^ which during subsequent infections allows them to be rapidly recruited to infected regions to allow localized antibody secretion^37,38,78^. Further H3-nanovax’s design allows for long-term local antigen depots^22,79^. This aspect of the vaccine may be critical in B_RM_ induction as prior studies have shown that local antigen encounter is necessary for B_RM_ formation after IAV infection^37^. Altogether, the ability of H3-nanovax to induce local GC B cells, B_RM_, and IgG/IgA antibodies in addition to systemic immune responses suggest that it may provide enhanced antibody mediated protection compared to currently approved IAV vaccines.

Despite the inclusion of differing IAV protein payloads, all three formulations of H3-nanovax induced lung parenchyma and CD11a^lo^CD44^+^ (i.e. airway-resident) CD4 and CD8 T cell responses, including T_RM_, that were of statistically equivalent magnitude. Interestingly, in addition to classical T_RM_, we observed and enumerated CD4 T cells that were CD69^+^ and CD103^+^ (Fig. 4c, g). It has been suggested that CD4 T cells co-expressing CD69 and CD103 in the lungs may be cytotoxic CD4 T cells and can contribute to direct viral control following IAV infection through class II MHC-restricted killing^58,80^. Regarding the CD8 T_RM_ populations, we observed that the CD69^+^CD103^+^ cells expressed a signature consistent with canonical lung CD8 T_RM_. However, as it has been noted that CD8 T_RM_ in other tissues (i.e. small intestine and liver) may be CD103+/−^59^, we also examined the T_RM_ signature of CD69^+^CD103^-^ CD8 T cells in the lung following nanovaccine administration. We observed that while this population expressed many of the markers associated with CD8 T_RM_ and may potentially contain CD8 T_RM_, the population was more heterogenous compared to the CD69^+^CD103^+^ cells, suggesting they may be a transitional population^50,59,60^. Specifically, it was observed that a substantial fraction of CD69^+^CD103^-^ cells were also CD49a^-^, therein not expressing a marker whose expression has been described to be as associated with CD8 T_RM_ cells^81,82^ (Supplementary Fig. 8). Importantly, for the first time, we show that in addition to lung parenchyma-based T cell responses, the IAV-nanovax platform induces CD11a^lo^CD44^+^ CD4 and CD8 T cells, a cell phenotype that is associated with airway-resident cells. These tissue-resident T cell responses have been previously shown to confer and enhance protection against subsequent infections by rapidly producing IFNγ upon re-exposures^61^. The observed increases in both total antigen-experienced and T_RM_ populations in the lung parenchyma and airways are promising considering the known protective role CD4 and CD8 T_RM_ and airway T cells have during IAV infections, especially in the context of heterosubtypic IAV infections^8,10,48–51^. The presence of these T cells after i.n. vaccination with all three H3-nanovax formulations was consistent with the protection we observed against a highly stringent lethal dose heterosubtypic H1N1 viral challenge (Fig. 5d–f) a model where anti-HA and anti-NA effector function blocking antibodies would not be predicted to contribute to protection.

The combined humoral and cellular immunity induced by our three formulations of H3-nanovax corresponded with protection against morbidity and mortality following homologous IAV H3N2 challenge (Fig. 5a–c). While vaccinated mice were protected, heterosubtypic H1N1 challenge revealed differences in the overall magnitude of protection between the three H3-nanovax formulations, with H3-nanovax (HA/NP) and H3-nanovax (NA/NP) vaccinated mice demonstrating the greatest reduction in mortality, with ~60+ % of mice surviving while >30% of H3-nanovax (NA/M1) vaccinated mice survived. In part this reduced level of protection observed with the H3-nanovax (NA/M1) may relate to the reduced GC B cell and BAL antibody-based immunity observed with this formulation, a result that could further highlight the importance of tissue-resident immunity to higher levels of protection. Alternatively, and/or in concert with these B cell/antibody changes, the differential protection of the three H3-nanovax formulations against heterosubtypic IAV, where antibodies against HA and NA would not be predicted to contribute to overall protection, may relate to the T cells generated against the antigens encapsulated in each H3-nanovax formulation. With regard to CD8 T cell responses against NP, NA, HA, and M1, the La Gruta group has characterized the magnitude of the CD8 T cell response on day 10 post H1N1 A/Puerto Rico/8/34 infection against NP_366-374_, NP_36-43_, NP_55-63_, NA_181-190_, HA_402-409,_ HA_389-399_, HA_41-49_, HA_308-316_, M1_181-190_, and M1_128-135_. In that study the NP_366-374_ (~7.5%) and NP_36-43_ (~1%) were by far the largest individual epitope responses against these proteins with total combined CD8 T cell responses to M1 (~0.6% total), HA (~1.2% total) and NA (~0.55%) epitopes of much smaller magnitudes^83^. Similarly, when the CD4 T cell response against A/New Caledonia/20/1999 H1N1 virus was examined in C57Bl/10 (I-A^b^) mice the NP specific CD4 T cells represented ~50% of the response, with T cells against NA representing the next largest subset and with responses to HA and M1 representing a minor portion. Therefore while we observed a similar magnitude of vaccine-specific CD4 and CD8 T cell responses after all three H3-nanovax vaccinations (Fig. 4), the differences in protection observed between H3-nanovax (NA/NP) and (HA/NP) vs. H3-nanovax (NA/M1) (Fig. 5) may relate to the relative abundance of total epitopes and/or conserved T cell epitopes found in the H3N2 proteins and the H1N1 challenge virus, with CD4 and CD8 epitopes in NP expected to be more prevalent in the C57Bl/6:A/PR/8 H1N1 challenge model utilized here. Of note, the C57Bl/6 NP_366_ epitope, which is an immunodominant epitope, in the H3N2 and H1N1 viruses, examined herein in Supplementary Fig. 10, differs by two amino acids (H3N2, ASNENMDAM; H1N1, ASNENMETM), suggesting that only a fraction of the NP_366_ T cells generated by the H3N2 NP protein may cross-react during the heterosubtypic IAV challenge. Further while total antigen-experienced and lung-tissue resident memory T cells are similar, our results suggest that NP_366_-specific T cells may localize to the lungs at an increased rate in the NA/NP vs HA/NP H3-nanovax group. This differential localization and lung-resident NP_366_ T_RM_ numbers could contribute to the increased protection we observe with the H3-nanovax NA/NP group after H1N1 virus challenge (Fig. 5). In contrast the M1_128_ epitope, which is known to generate a much smaller T cell response, consistent with our findings in Supplementary Fig. 10, is conserved between the H3N2 and H1N1 viruses in C57Bl/6 mice. Despite these differences in the degree of protection, all three H3-nanovax formulations did confer protection against the high dose lethal H1N1 heterosubtypic challenge suggesting a role for protective T cells in reducing viral titers and disease following heterosubtypic IAV challenge (Fig. 6b). Further, given this protection and the fact that we have observed protection against heterosubtypic infections in outbred mice (i.e., a model with diverse MHC haplotypes) with our H1-nanovax^24^, it would be expected that the three H3-nanovax formulations either alone or administered in combination may generate the multi-focused T cell response that is necessary to provide broad-based protection of outbred populations against heterosubtypic IAV viruses. Such T cell mediated immunity along with non-neutralizing antibodies against NP and M1 could provide protection even in the face of challenges with highly pathogenic heterosubtypic IAV strains.

In conclusion, we have demonstrated that H3-nanovax (HA/NP), (NA/NP), and (NA/M1) induce humoral and cellular immune responses both systemically and in the lung mucosa/airways; these include lung GC B cells and B_RM_ associated with systemic and local whole virion and protein-specific IgG and IgA. The H3-nanovaccines also generated systemic and mucosal airway and lung parenchyma antigen-experienced CD4 and CD8 T cells, including lung tissue-resident memory. Together the presence of these immune responses corresponded with protection against both lethal homologous and heterosubtypic IAV challenge. Moreover, combined with the flexibility and utility of the IAV-nanovax platform, H3-nanovax represents a significant advance in our understanding of how to confer optimal immunity and broad-based protection against multiple IAVs.

## Methods

### IAV-nanovax synthesis

Monomers based on 1,8-bis(*p*-carboxyphenoxy)-3,6-dioxoctane (CPTEG) and 1,6-bis(*p*-carboxyphenoxy)hexane (CPH) were synthesized as described previously^84,85^. Using these monomers, 20:80 CPTEG:CPH copolymer was synthesized using melt polycondensation for ~6 h, as described^85^. The final copolymer composition, purity, and molecular weight of the copolymer were characterized using ^1^H NMR (DXR 500, Bruker, Billerica, MA). Next, 20:80 CPTEG:CPH nanoparticles containing 2 wt.% CpG (ODN 1668, Invivogen, San Diego, CA) adjuvant as well as 1 wt.% H3N2 HA (Sino Biological, Beijing, China, Catalog # 40116-V08B) and 1 wt.% NP (Sino Biological, Catalog # 40207-V08B), 1 wt.% H3N2 NA (Sino Biological, Catalog # sino) and 1 wt.% NP, or 1 wt.% NA and 1 wt.% H3N2 M1 (Sino Biological, Catalog # 40215-V07E) were synthesized via solid-oil-oil double emulsion^86^. All influenza virus proteins were purchased from Sino Biological. The HA (aa1-530-his tag; >95% purity, ~65 kDa) and NP (aa1-498-his tag; >90% purity, ~54.4 kDa) proteins were from baculovirus infected insect cells, the NA (aa36-469-his tag; >90% purity, ~76.8 kDa) was produced in HEK293 cells, and M1 (aa1-252-his tag; >85% purity, ~35 kDa) purified from *E. coli*. The HA had a reported ability to agglutinate guinea pig RBCs at a HA titer of 0.5–5 µg/mL for 1%RBCs. Briefly to synthesize the vaccine, the protein antigens were dialyzed to nanopure water and lyophilized overnight. The 20:80 CPTEG:CPH copolymer, along with the protein antigens and CpG, was dissolved at a polymer concentration of 20 mg/mL in methylene chloride. The solution was sonicated for 30 s and then precipitated into chilled pentane (at a methylene chloride:pentane ration of 1:250). The resulting nanoparticles were collected via vacuum filtration and scanning electron microscopy (FEI Quanta 250, FEI, Hillsboro, OR) was used to characterized morphology and size. The average size distribution of each formulation was determined by analyzing ~500 particles of each scanning electron micrograph using ImageJ software (ImageJ 1.53t, National Institutes of Health, Bethesda, MD). The antigen encapsulation efficiency of each H3-nanovax formulation was determined by incubating the particles in 40 mM sodium hydroxide to rapidly degrade the polymer and release the encapsulated proteins. The released proteins were then quantified via a micro bicinchoninic acid assay (microBCA, Thermo Fisher Scientific, Waltham, MA).

### Primary structural analysis of IAV proteins released from polyanhydride nanoparticles

Encapsulated proteins were released by resuspension of the particles in 300–400 μL of PBS and incubation at 37 °C overnight. Suspensions were then pelleted, and supernatant was collected and quantified for protein content via micro-bicinchoninic acid (BCA) assay (Pierce, Rockford, IL). Subsequently, to confirm the integrity of the primary structure of the encapsulated protein, released samples were analyzed via sodium dodecyl sulfate polyacrylamide gel electrophoresis (SDS-PAGE). To this end, liberated proteins from the three H3-nanovax formulations, purified HA, NA, NP, or M1 protein (source: original input Sino proteins), ECL DualVue^TM^ Western Blotting Ladder (Cytiva, Marlborough, MA), and/or precision Plus Dual Protein Standard (Biorad Laboratories) were individually combined with 4 x LDS Sample Buffer (Invitrogen) and ddH20 to a volume of 30 μL. Each sample was loaded into individual wells of a Novex 4–20% Tris-Glycine Plus WedgeWell^TM^ Gel (Thermo Fisher Scientific). Electrophoresis was performed for 30–40 min at 125 V at 4 °C in MOPS SDS buffer (Invitrogen). Gels were transferred onto a PVDF membrane (Bio-Rad Laboratories, Hercules, CA) in a mini blot module (Thermo Fisher Scientific) at 4 °C at 20 V for 1 h. Following transfer, membranes were blocked in 5% milk for 1 h, washed, and incubated with primary antibody (anti-HA: Sino Catalog #86001-RM01; anti-NA (N2): Sino, Catalog #40040-MM02; anti-NP: Invitrogen, Catalog #MA5-42364; anti-M1: Invitrogen, Catalog #PA5-32253) against their respective protein overnight at 4 °C. Following, membranes were washed and incubated with goat anti-rabbit horseradish peroxidase (HRP, Thermo Fisher Scientific) and ECL DualVue^TM^ Western Blotting Marker S-Protein-HRP for 1 h, washed, and the western was developed with an ECL substrate (Bio-Rad Laboratories) for 5–10 min. Images were obtained on a ChemiDoc MP Imager (Bio-Rad Laboratories). Western blot images were analyzed in Microsoft Powerpoint (Redmond, WA) where blot photos were cropped and corrected using sliders for sharpness, brightness, and saturation.

### Mice, vaccination, and influenza virus infections

Wild type female C57BL/6 mice were bred, housed, and maintained in the University of Iowa (Iowa City, IA) animal care facilities. All procedures were performed on matched mice, were approved by the Institutional Animal Care and Use Committee of the University of Iowa and comply with the NIH Guide for Care and Use of Laboratory Animals. 8–12 week-old 20–22 g mice were randomly assigned into groups for each experiment. Prior to i.n IAV-nanovax vaccinations and IAV infections, mice were anesthetized with isoflurane. For each IAV-nanovax i.n. administration, mice received 500 μg of one of three formulations of H3-nanovax (containing a total of 10 μg) CpG1668 and 5 μg of each IAV protein; (i.e., HA + NP, NA + NP, or NA + M1) in 50 μL of PBS. Mice were subsequently boosted, receiving a second i.n. dose of the respective H3-nanovax formulation 14 days after the initial priming. As a control, groups of mice were administered nanoparticles containing CpG without the IAV proteins (i.e., CpG only). For IAV challenge, mice were infected i.n. with a 1108 TCIU dose of A/Puerto Rico/8/34 (H1N1) or a 390 TCIU dose of A/Hong Kong/1/68 (H3N2) in 50 μL Iscove’s Modified Dulbecco’s Medium. Following IAV infection, body weight, airway restriction (Penh), and survival were monitored daily for 14 days. Penh was measured using unrestrained whole-body plethysmography (Buxco Electronics, Wilmington, NC) recording volume and pressure changes over 5 min. Mice were euthanized via cervical dislocation upon reaching ≤ 70% of their starting weight.

### 50% Tissue Culture Infectious Dose (TCID50) assay for viral titers

Briefly, Dulbecco’s Modified Eagle Medium (DMEM) (Thermo Fisher Scientific, Waltham, MA) containing 50 µg/mL gentamicin (Thermo Fisher Scientific, Waltham, MA), 100 µg/mL penicillin/streptomycin (Thermo Fisher Scientific, Waltham, MA), and 2.5 µg/mL amphotericin B (Thermo Fisher Scientific, Waltham, MA) were added to the wells of a 96-well plate. Subsequently, homogenized lung samples were added to the top well in triplicate at a 1:10 dilution and serially diluted at 1:10. 100 µL 2.5 × 10^5^ Maden-Darby Canine Kidney (MDCK) cells were then added to the wells and incubated overnight in a humidified, 37 °C, 5% CO_2_ incubator. Following overnight incubation, the DMEM from the wells was discarded and 200 µL of antibiotic containing DMEM + 0.0002% trypsin (Thermo Fisher Scientific, Waltham, MA) was added to the wells and incubated at 37 °C for a further 3 days. On day 5 following initial sample addition, each well then received 50 µL of 0.5% chicken RBC suspension (Rockland Immunochemicals, Philadelphia, PA) and incubated at 4 °C for 1 h after which agglutination patterns were recorded and the TCID_50_ was calculated for each group.

### Intravascular staining to determine cellular localization

To discriminate between circulating and tissue-resident cell populations, mice were administered 1 μg of fluorophore-conjugated rat anti-mouse CD45.2 (clone 104; BioLegend, San Diego, CA) in 200 μL of PBS by retroorbital intravenous injection 3 min prior to harvest as previously described^36^.

### Serum, bronchial alveolar lavage, and cell isolation

Prior to euthanasia, blood was collected in non-heparinized capillary tubes (Fisher Scientific) for serum collection. Blood samples were left at room temperature for 30 min, centrifuged at 16,000 × g for 20 min, and then collected and stored at −20 °C until analysis.

Bronchial alveolar lavage (BAL) fluid was collected using a protocol modified from^87^. Briefly, the tracheae were cannulated with a 22-gage catheter tube (attached to a 5cc syringe) and then washed once with 1 mL of sterile PBS. Samples were stored at −20 °C until analysis. For preparation of cells, lungs and mediastinal lymph nodes were harvested after the collection of BAL fluid, digested for 30 min at 37 °C in media containing 1 mg/mL Collagenase (Type 11, Sigma Aldrich, St. Louis, MO) and 0.02 mg/mL DNase-I (MP Biomedicals, Santa Ana, CA), homogenized in gentleMACS™ C tubes utilizing the gentleMACS™ Octo Dissociator program m_lung_02_01 (Miltenyi Biotec, Gaithersburg, MD), and subsequently strained through 70 µm MACS^®^ SmartStrainers (Miltenyi Biotec) into single cell suspensions.

### IAV-specific whole virus Enzyme-linked Immunosorbent Assays (ELISAs)

Total IAV-specific IgG and IgA antibody against whole IAV H3N2 A/Hong Kong/1/68 live virus was measured as previously described^88^. Briefly, wells were coated with ~3.2 × 10^5^ TCIU_50_ of virus, blocked with 1% bovine serum albumin for 1 h, washed, and then blotted dry. Serum or BAL samples were added to the top well in triplicate at a 1:50 or 1:4 dilution in 200 μL/well, respectively. Samples were serially diluted at 1:2 and incubated at 37 °C for 2 h. Plates were washed, blotted dry, and then IAV-specific antibody was detected using the following antibodies: Biotin-SP (long spacer) AffiniPure Goat Anti-Mouse IgG (Catalog # 115-065-071), (Jackson Immunoresearch Laboratories, West Grove, PA) or biotinylated goat anti-mouse IgA (Bio-Rad, Hercules, CA) followed by peroxidase conjugated-streptavidin (Jackson Immunoresearch Laboratories) and TMB substrate (Abcam, Boston, MA). Reaction was stopped using 0.16 M H_2_SO_4_ and optical densities were measured at 450 nm using a SpectraMax M5 Multi-mode microplate reader from Molecular Devices (Sunnyvale, CA).

### Hemagglutinin inhibition assays

HAI assays using mouse serum and BAL were performed as previously described^89^. Briefly, sera and BAL were inactivated by heating at 56 °C for 30 min and then adsorbed in a chicken red blood cell (CRBC) suspension for 30 min at different concentrations: serum was absorbed in 1% CBRC at 1:5 and BAL was absorbed in 10% CRBC at 1:2. CRBCs were pelleted, sera and BAL were recovered, and were serial diluted in 96-well round-bottom plates that were then incubated with four hemagglutination units of stock A/Hong Kong/1/68 (H3N2) virus per well for 30 min. Each well then received 1% CRBC suspension and HAI titer was measured after a 30 min incubation.

### Neuraminidase inhibition assays

Neuraminidase inhibition^46^ assays using mouse serum and BAL were performed as previously described^90^. Serum and BAL were inactivated by heating at 56 °C for 30 min. Briefly, 70 μL of serum or BAL were 2-fold serially diluted in 96-well round-bottom plates. 5.85 × 10^6^ TCIU of H3N2 A/Hong Kong/1/68 virus in assay buffer containing 0.1% NP-40 detergent solution was added to each well except for blank control wells and incubated at room temperature for 30 min. Next, 50 μL from each well was then transferred into a clear 96-well flat-bottom plate and each well received 50 μL 30 μM 2′-(4-Methylumbelliferyl)-α-D-*N*-acetylneuraminic acid (MUNANA, excitation wavelength = 317 nm) working solution. Plates were covered and incubated for 1 h at 37 °C. Following incubation, stop solution (0.824 M NaOH) was added to terminate the reaction and plates were read at 460 nm using a SpectraMax M5 Multi-mode microplate reader where OD were converted to IC_50_ and IC_25_ based on activity relative to NA-activity in the absence of antibody.

### Nucleoprotein (NP) and Matrix Protein 1 (M1)-specific ELISAs

Total NP-specific or M1-specific IgG and IgA antibody against whole NP (Sino Biological, Beijing, China; Catalog # 40208-V08B, Baculovirus Insect cells) or whole M1 (Sino Biological, Beijing, China; Catalog # 40215-V07E, *E. coli*) derived from H3N2 IAVs A/Hong Kong/1/68 and A/Aichi/2/68 respectively or control SARS-CoV-2 spike protein S1 subunit (Sino Biological; Catalog #40150-V05H1) was measured as previously described^88^. Briefly, wells were coated with 2 μg/mL of NP or M1, blocked with 1% bovine serum albumin and ChromPure Goat IgG whole molecule (Jackson Immunoresearch Laboratories, West Grove, PA) and ChromPure Human IgG whole molecule (Jackson Immunoresearch Laboratories, West Grove, PA) diluted at 1:500 within the blocking solution. After blocking for 1 h, plates were washed and then blotted dry. Serum or BAL samples were added to the top well in triplicate at a 1:50 or 1:4 dilution in 200 μL/well, respectively. Samples were serially diluted at 1:2 and incubated at 37 °C for 2 h. Plates were washed, blotted dry, and then NP or M1-specific antibody was detected using the following antibodies: Biotin-SP (long spacer) AffiniPure Goat Anti-Mouse IgG (Jackson Immunoresearch Laboratories) or biotinylated goat anti-mouse IgA (Bio-Rad) followed by peroxidase conjugated-streptavidin (Jackson Immunoresearch Laboratories) and TMB substrate (Abcam). The reaction was stopped using 0.16 M H_2_SO_4_ and optical densities were measured at 450 nm using a SpectraMax M5 Multi-mode microplate reader.

### Antibody staining for flow cytometry

Single-cell suspensions (1 × 10^6^ cells) from lungs and lung-draining lymph nodes (dLN) were blocked with 2% rat serum for 30 min at 4 °C. Following blocking, cells were stained with the following antibodies to identify CD4 and CD8 T cell subsets: 1:500 rat anti-mouse CD62L (MEL-14; Biolegend, cat #: 104430), 1:500 rat anti-mouse CD4 (GK1.5; BioLegend, cat #: 100480), 1:500 rat anti-mouse CD8α (53-6.7; BioLegend, cat #: 569185), 1:250 rat anti-mouse CD49d (R1-2; Biolegend, cat #: 103606), 1:250 rat anti-mouse CD11a (M17/4; BD Biosciences, San Jose, CA, cat #: 741919), 1:100 rat anti-mouse CD103 (M290; BD Biosciences, cat #: 121433), 1:100 rat anti-mouse CD69 (H1.2F3; eBioscience, cat #: 104527), 1:100 rat anti-mouse Eomes (Dan11mag; Invitrogen, cat #: 14-4875-82), 1:250 hamster anti-mouse/rat CD49a (Ha31/8; BD Biosciences, cat #: 562115), hamster anti-mouse 1:100 CXCR3 (CXCR3-173; Biolegend, cat #: 126516), and 1:100 anti-mouse CX3CR1 (SA011F11; Biolegend, cat #: 149031). Antigen experienced T cells were identified via expression of surrogate markers as previously described^53,54^. Briefly, CD11a^hi^CD49d^+^ expression was utilized to identify antigen-experienced CD4 T cells (CD19^-^CD4^+^CD8α^-^CD45.2ivAb^-^), while CD11a^hi^CD44^+^ expression was utilized to quantify antigen-experienced CD8 T cells (CD19^-^CD8α^+^CD4^-^CD45ivAb^-^) in the lungs and airways (see Supplementary Fig. 2). CD44^+^CD45.2ivAb^-^ CD4 and CD8 T cells were analyzed for tissue localization to the airway (CD11a^lo^CD44^+^) or lung parenchyma (CD11a^hi^CD44^+^) as previously described^63^. Vaccine specific CD8 T cells were identified using A/Hong Kong/1/68 H2-D^b^ NP_366-374_ (ASNENMDAM) and H2-K^b^ M1_128-135_ (MGLIYNRM) tetramers diluted 1:100 that were produced from monomers generated similar to as previously described^91^. To identify B cell subsets, cells were stained with rat anti-mouse 1:500 CD19 (1D3; BD Biosciences, cat #: 115545), 1:250 rat anti-mouse B220 (RA3-6B2; BioLegend, cat #: 558108), 1:250 rat anti-mouse IgM (B7-6; eBioscience, cat # 46-6526-42), 1:250 rat anti-mouse Fas (Jo2; BioLegend, cat #: 565605), 1:100 rat anti-mouse GL7 (GL7; Biolegend, cat #: 144610), 1:100 rat anti-mouse CD38 (90; BD Biosciences, cat #: 746476), 1:100 hamster anti-mouse CD69 (H1.2F3; Biolegend, cat #: 104527), 1:100 rat anti-mouse CD73 (TY/11.8; Biolegend, cat #: 127208), and 1:100 hamster anti-mouse CXCR3 (CXCR3-173; Biolegend, cat #:149031). Cells were then fixed with BD FACS™ Lysing Solution per manufacturer’s instructions and resuspended in PBS. Data were acquired on a LSRII (BD Biosciences) or Cytek Aurora (Cytek Biosciences, Fremont, CA) and analyzed using FlowJo software (Tree Star, Ashland, OR).

### Statistical analyses

Experiments were repeated at least twice unless noted otherwise. Comparisons between more than two groups at different time points were analyzed using two-way ANOVA with Tukey’s multiple comparison *post-hoc* test. For comparisons between more than two groups at a single time point, a D’Agostino and Pearson normality test was performed to establish normality. Data that failed normalcy were analyzed using a Kruskal–Wallis ANOVA with a Dunn’s multiple comparison *post-hoc* test. Data that passed normalcy were analyzed using a one-way ANOVA with a Tukey’s multiple comparison post-hoc test. A *P* ≤ 0.05 was considered significant.

### Reporting summary

Further information on research design is available in the Nature Research Reporting Summary linked to this article.

### Supplementary information

Supplementary Figures

Reporting summary

## Data Availability

All data generated or analyzed from which conclusions are drawn in this manuscript are available within the paper and Supplementary Information.
