# Supplementary material for: Polyanhydride nanovaccine against H3N2 influenza A virus generates mucosal resident and systemic immunity promoting protection
Source: NPJ Vaccines. 2024 May 31;9:96. doi: 10.1038/s41541-024-00883-3 (PMC11143372; doi:10.1038/s41541-024-00883-3)
Supplement: Supplementary file 1 — Supplementary Figures [file 41541_2024_883_MOESM1_ESM.pdf]

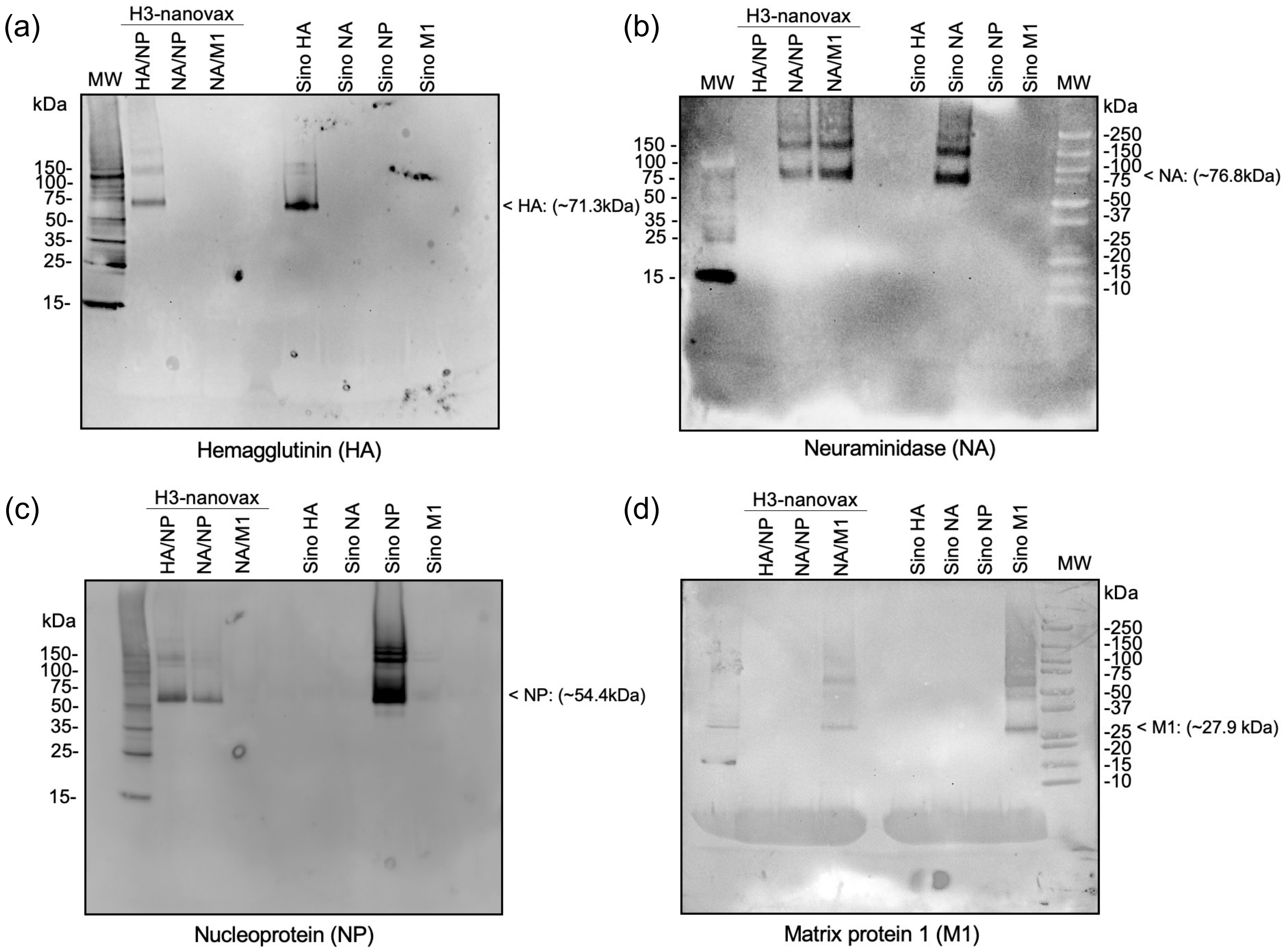


**Supplementary Fig. 1. The IAV proteins released from H3-nanovax formulations remain structurally intact**. Proteins liberated from the three H3-nanovax formulations and the input purified HA, NA, NP, or M1 proteins (Sino) were examined by SDS PAGE under reducing conditions. Gels were transferred onto a PVDF membrane, membranes blocked in 5% milk for 1 hr, washed, and incubated with primary antibody against their indicated protein overnight at 4°C. Membranes were then washed and incubated with goat anti-rabbit horseradish peroxidase (HRP) as well as ECL DualVue™ Western Blotting Marker S-Protein-HRP to reveal molecular weight markers for 1-2 hrs, washed, and then reveled with an ECL substrate for 5-10 min. Images were obtained on a ChemiDoc MP Imager (Bio-Rad Laboratories). SDS-Page gels against hemagglutinin (HA: antibody 86001-RM01) (a), neuraminidase (NA: antibody 40040-MM02) (b), nucleoprotein (NP: antibody MA5-42364) (c), and matrix protein 1 (M1: antibody PA5-32253) (d). The predicted MW: Molecular weight kDa (kilodaltons) is indicated.

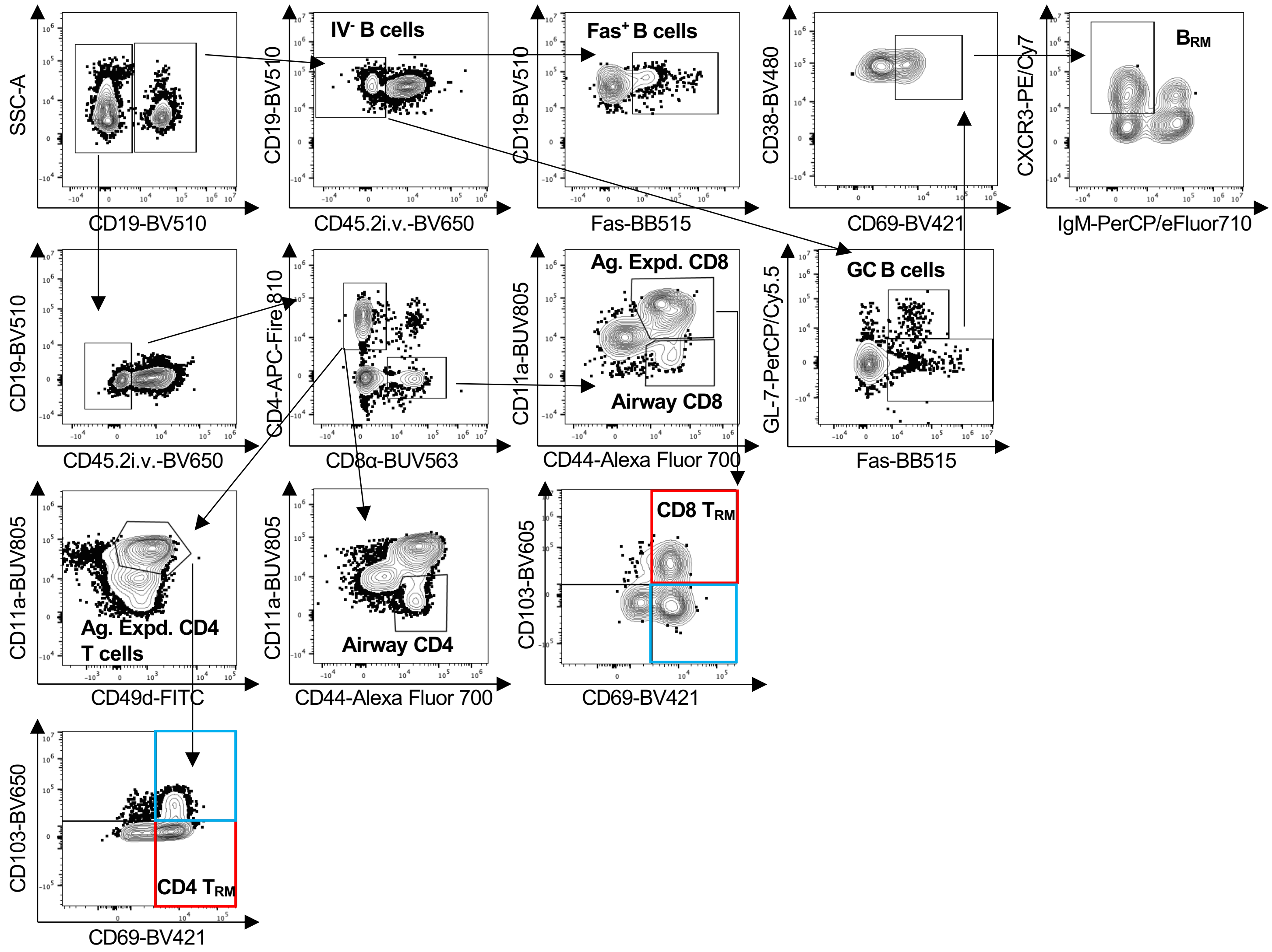


**Supplementary Fig. 2. Lymphocyte Gating Tree.** Lymphocyte Gating Tree. C57BL/6 mice were vaccinated i.n. with 500 µg of H3-nanovax encapsulating CpG + either 1% each HA + NP, M1 + NA, or NA + NP or left unvaccinated (naïve). At 45- or 50- days post vaccination lungs and draining lymph nodes were harvested and stained for expression of markers of interest. B and T cells were segregated based on CD19, CD4, and CD8α expression. B cells within the lung vs those in the circulation were further identified based on CD45.2i.v.Ab$^-$. B cells within the lung were further subset into FAS$^+$ B cells (CD45.2i.vAb$^-$CD19$^+$Fas$^+$) and Germinal Center (GC) B cells (CD45i.vAb$^-$CD19$^+$ Fas$^+$GL7+). Fas$^+$GL7$^-$ B cells were further subset into resident memory B cells (B$_{RM}$; CD45i.vAb$^-$CD19$^+$ Fas$^+$GL7$^-$CD38$^+$CD69$^+$IgM$^-$CXCR3$^+$). T cell populations in CD19$^-$ cells were gated based on CD4 or CD8α expression and then further subset into antigen-experienced populations based on CD11a, CD49d, CD44 expression (AgExp CD4; CD4$^+$CD11a$^{hi}$CD49d$^+$ and AgExp CD8; CD11a$^{hi}$CD44$^+$. Airway resident T cells were identified on the basis of CD11a expression (airway CD4/CD8 T cells; CD11a$^{lo}$CD44$^+$). Antigen-experienced lung-resident memory T$_{RM}$ populations of CD4 and CD8 T cells were determined on the basis of CD45.2i.v.Ab$^-$, CD69 and CD103 expression.

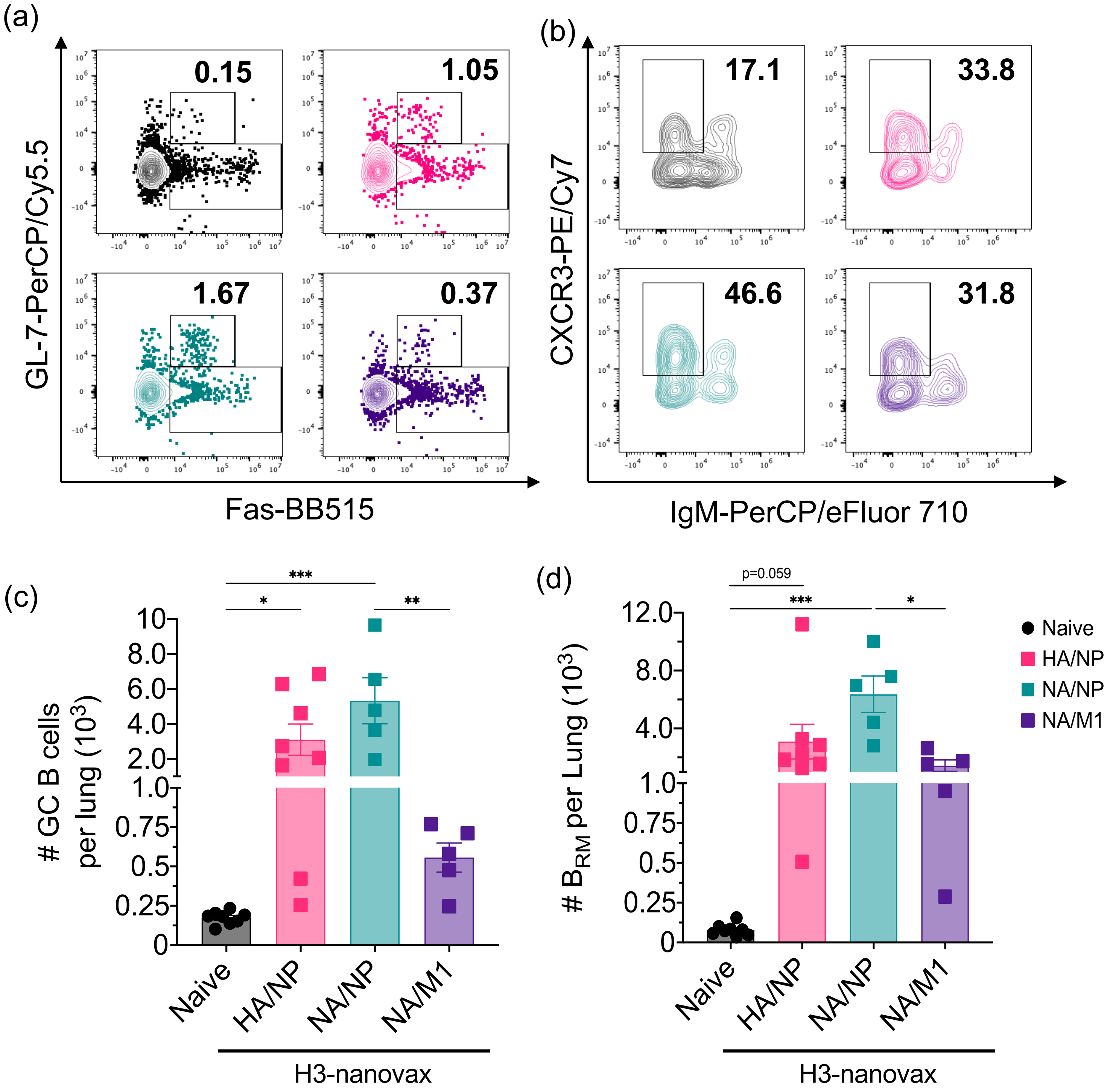


**Supplementary Fig. 3. Germinal Center B cells and $B_{RM}$ in the lungs following H3-nanovax. Supplemental Figure 2. Germinal Center B cells and $B_{RM}$ in the lungs following H3-nanovax.** C57BL/6 mice were vaccinated i.n. with 500 μg of H3-nanovax (HA/NP; Pink), (NA/NP; Blue), (NA/M1; Purple), or left unvaccinated (naïve). Representative flow plots (a, b) and enumeration (c, d) of lung resident germinal center (GC) B cells (CD45i.vAb$^{-}$CD19$^{+}$ Fas$^{+}$GL7$^{+}$, shown are CD45i.v.Ab$^{-}$CD19$^{+}$ gated cells) (a, c), and resident memory B cells ($B_{RM}$) (CD45i.vAb$^{-}$CD19$^{+}$ Fas$^{+}$GL7$^{-}$CD38$^{+}$CD69$^{+}$IgM$^{-}$ CXCR3$^{+}$, shown are CD45i.vAb$^{-}$CD19$^{+}$ Fas$^{+}$GL7$^{-}$CD38$^{+}$CD69$^{+}$ cells) (b, d) at 45 or 50 days post vaccination. Error bars, mean ± s.e.m. Data representative of one (NA/NP, NA/M1: n= 5 mice/group) or two (Naïve, HA/NP: n= 4 mice/group) independent experiments. * P< 0.05, ** P< 0.01 (One-way ANOVA with Tukey's multiple comparisons test).

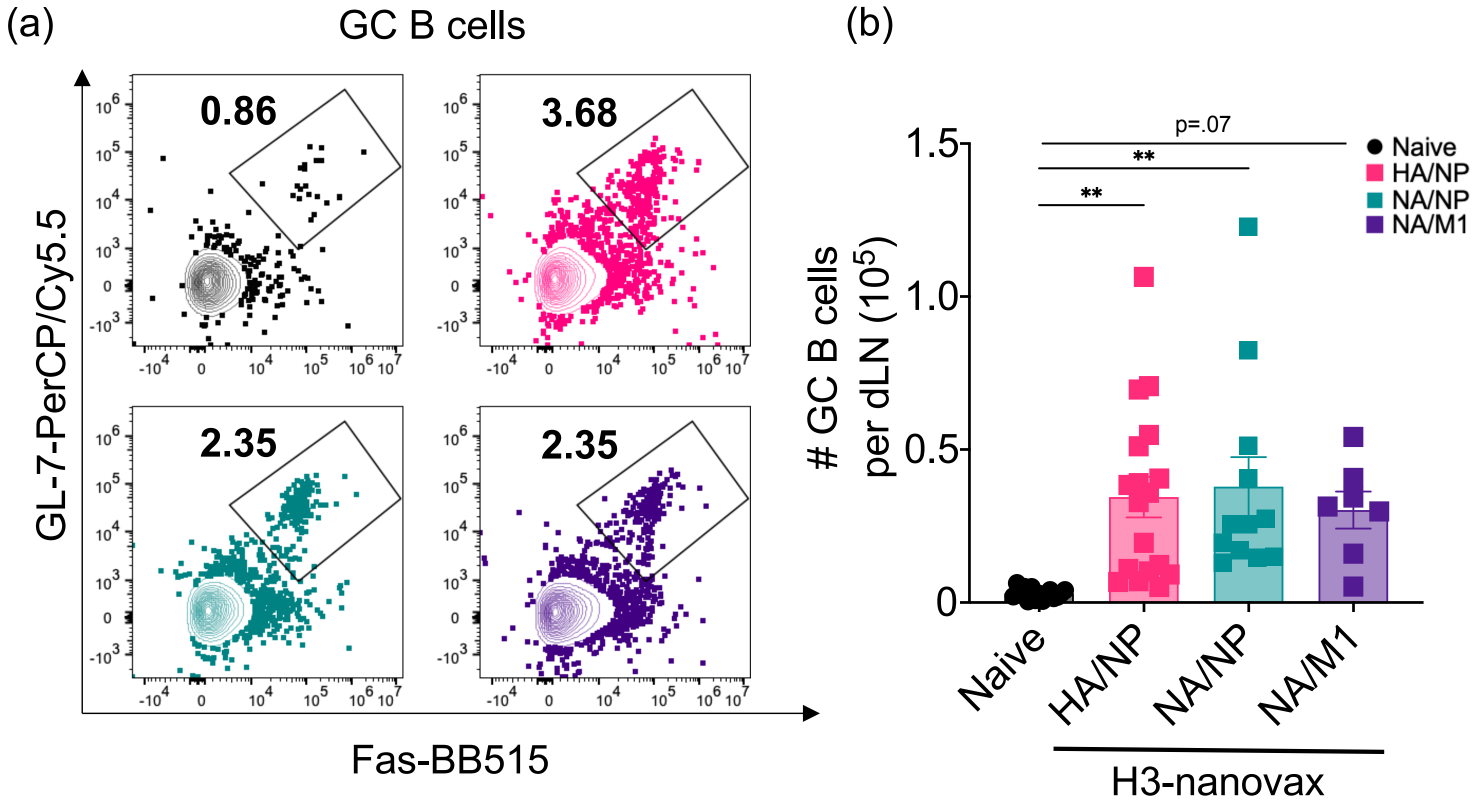


**Supplementary Fig. 4. H3-nanovax formulations induce lymph node-resident Germinal Center B cells responses.** C57BL/6 mice were vaccinated i.n. with 500 µg of H3-nanovax (HA/NP; Pink), (NA/NP; Blue), (NA/M1; Purple), or left unvaccinated (naïve). Representative flow plots (a) and enumeration (b) of lymph node resident germinal center (GC) B cells ($Fas^{+}GL7^{+}$) at 45- or 50-days post vaccination. Shown are $CD45i.vAb^{-}CD19^{+}$ gated cells. Error bars, mean ± s.e.m. Data representative of two (NA/M1: n= 3-4 mice/group) or three (Naïve, HA/NP, and NA/NP: n= 4-5 mice/group) independent experiments. * P< 0.05, ** P< 0.01 (One-way ANOVA with Tukey's multiple comparisons test).

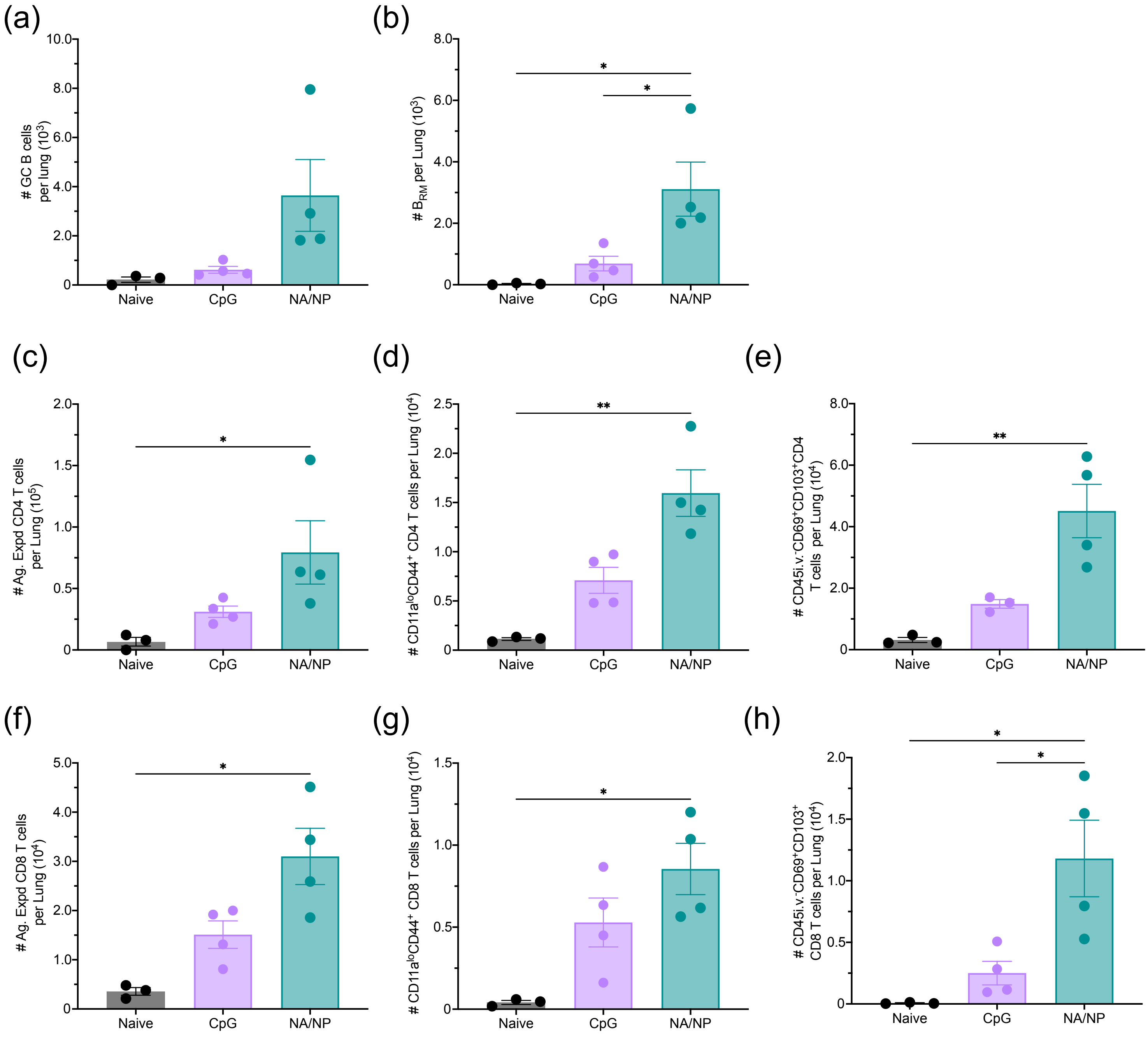


**Supplementary Fig. 5. CpG-nanovax does not induce significant immunity relative to unvaccinated mice.** C57BL/6 mice were vaccinated as described in Fig. 2. 32 days following prime vaccination, lungs were harvested and GC and $B_{RM}$ (a, b) B cell responses were enumerated. Total numbers of antigen experienced lung ($CD11a^{hi}CD44^{+}$) (c, f), airway ($CD11a^{lo}CD44^{+}$) (d, g), and CD4 ($CD69^{+}CD103^{-}$, e) and CD8 ($CD69^{+}CD103^{+}$,h) Trm T cells were also enumerated. Error bars, mean ± s.e.m. Data representative of one n= 3-4 mice/group) independent experiment. * $P< 0.05$, (One-way ANOVA with Tukey's multiple comparisons test).

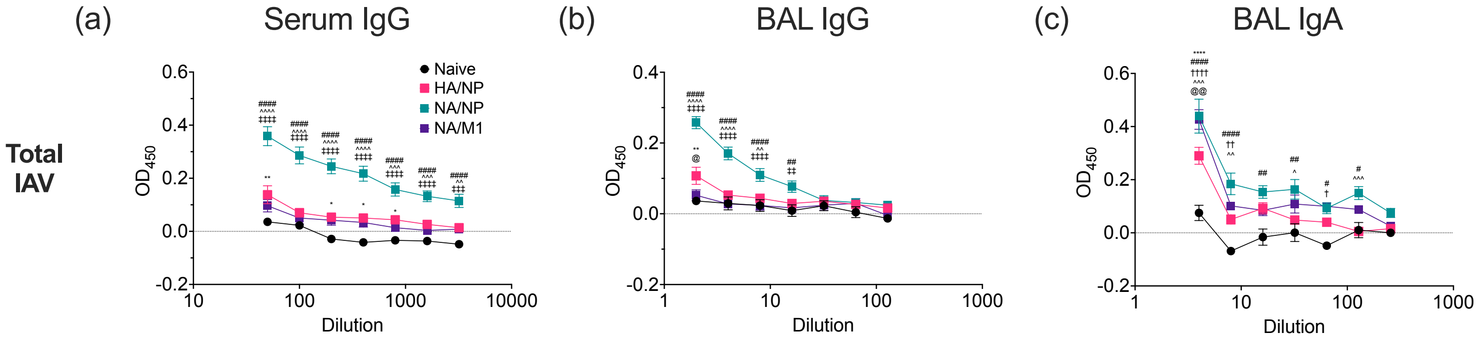


**Supplementary Fig. 6. H3-nanovax encapsulating IAV proteins induces payload targeted systemic and lung-local IAV-specific antibody responses.** C57BL/6 mice were vaccinated with H3-nanovax encapsulating CpG + either HA/NP (Pink), NA/NP (Blue), NA/M1 (Purple), or left unvaccinated (naïve). At 45 days post-vaccination, serum and BAL were collected and total IAV H3N2 A/Hong Kong/1/68 (i.e., virion specific) serum and BAL IgG (a, b) and BAL IgA (c) were quantified via ELISA. Error bars, mean ± s.e.m. Data representative of two independent experiments with n = 4 mice/group. (a-c) HA/NP vs. naïve: * P< 0.05, ** P< 0.01, *** P< 0.001, **** P< 0.0001; NA/NP vs. naïve: # P< 0.05, ## P< 0.01, ### P< 0.001, #### P< 0.0001; NA/M1 vs. naive: † P< 0.05, †† P< 0.01, ††† P< 0.001, †††† P< 0.0001; HA/NP vs. NA/M1: @ P< 0.05, @@ P< 0.01, @@@ P< 0.001, @@@@ P< 0.0001, NA/NP vs. NA/M1: ǂ P< 0.05, ǂǂ P< 0.01, ǂǂǂ P< 0.001, ǂǂǂǂ P< 0.0001, (Two-way ANOVA with Tukey's multiple comparison test). (g, h) **** P< 0.0001 (One-way ANOVA with Tukey's multiple comparisons test).

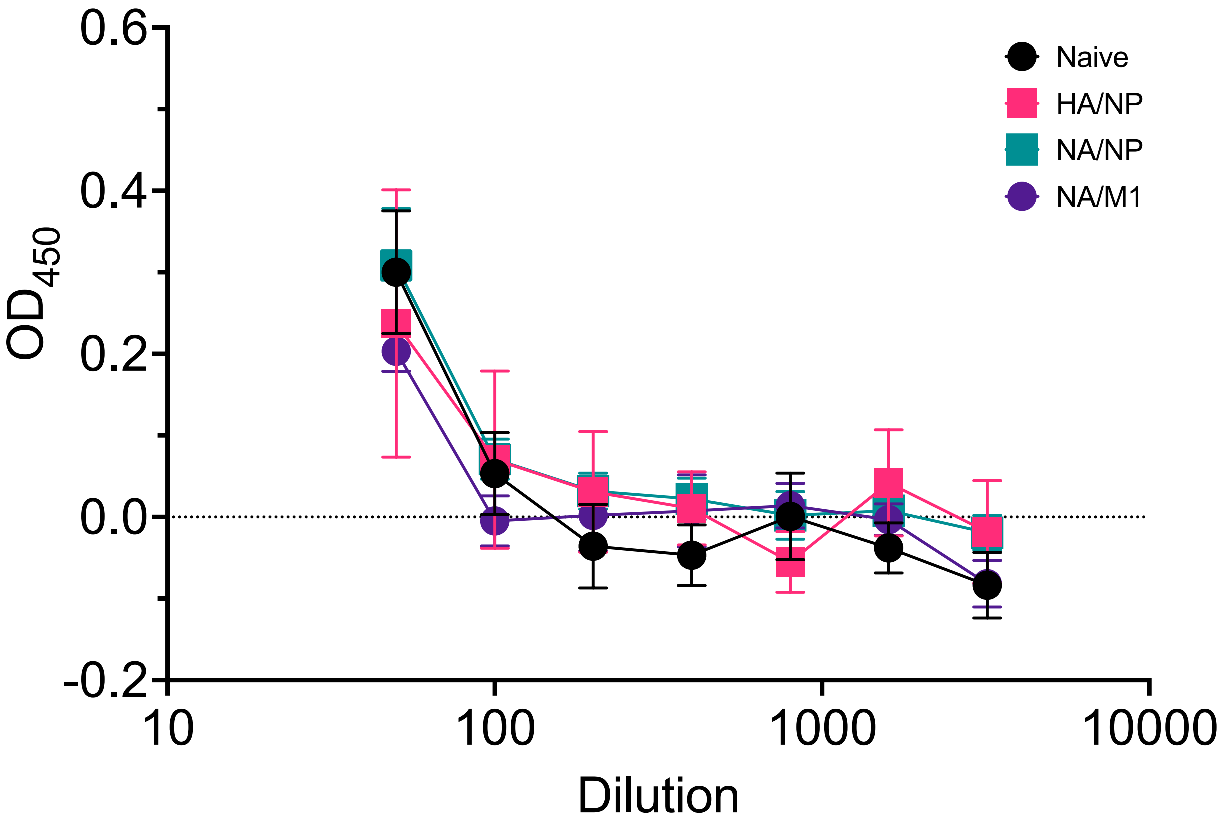


**Supplementary Fig. 7. IAV-nanovax induced antibody is specific to the viral protein component rather than the His-tag.** C57BL/6 mice were vaccinated as described in Fig. 2. At 45- or 50- days post vaccination, serum and BAL were collected and ELISA and total anti-His-SARS-CoV-2 S1 subunit specific IgG was quantified to determine whether His-tags on the proteins utilized in H3-nanovax formulations elicited His-reactive antibody. Error bars, mean ± s.e.m. Dotted line = 0. Data representative of two independent experiments with n = 4 mice/group. Two-way ANOVA with Tukey's multiple comparison test

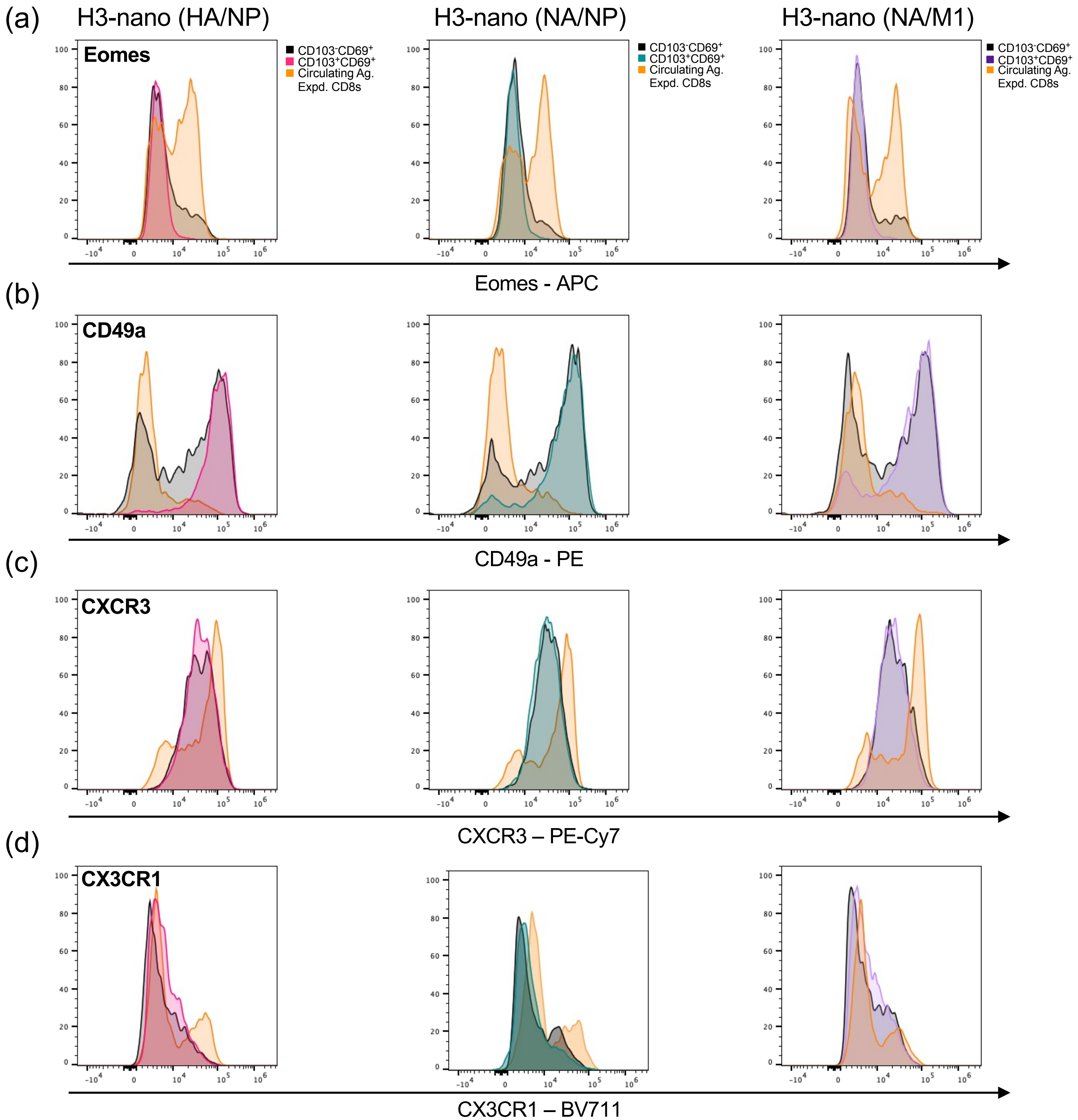


**Supplementary Fig. 8. H3-nanovax formulation generated antigen-experienced CD103$^{+}$CD69$^{+}$ CD8 T cells exhibit a uniform T$_{RM}$ phenotype relative to CD103$^{-}$ and circulating counterparts.** C57BL/6 mice were vaccinated as described in Figure 2. 32 days following prime vaccination, lungs were harvested, and the phenotype of vaccine generated CD103$^{+}$CD69$^{+}$ (HA/NP: Pink, NA/NP: Blue, NA/M1: Purple) and CD103$^{-}$CD69$^{+}$ (Gray) antigen-experienced CD8 T cells in the lung interstitium was determined using established T$_{RM}$ markers Eomesodermin (Eomes) (a), CD49a (b), CXCR3 (c), and CX3CR1 (d). Circulating antigen-experienced CD8 T cells (i.e. CD45.2i.v.Ab$^{+}$: Orange) served as a control. Data representative of one (n= 4-5 mice/group) independent experiment.

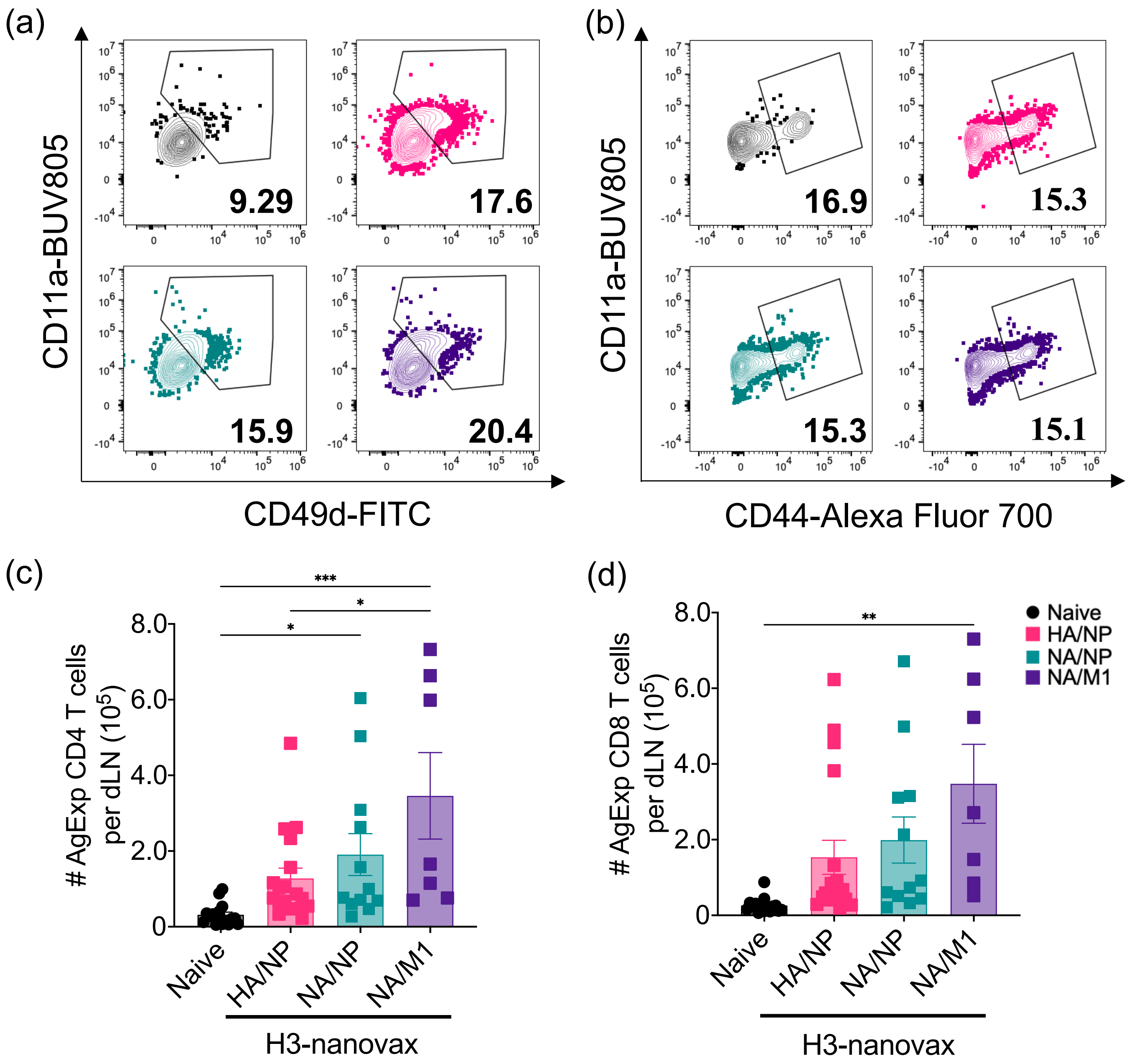


**Supplementary Fig. 9. H3-nanovax formulations induce lymph node-resident T cell responses.** C57BL/6 mice were vaccinated (H3-nanovax (HA/NP; Pink), (NA/NP; Blue), (NA/M1; Purple), or left unvaccinated (naïve)) as described in Fig. 2. Representative flow plots (a, b) and enumeration (CD4: c; CD8: d) of antigen-experienced CD4 (c, CD11a$^{hi}$CD49d$^{+}$) and CD8 (b, CD11a$^{hi}$CD44$^{+}$) T cells enumerated at 45- or 50- days post vaccination. Shown are CD4$^{+}$CD11a$^{hi}$CD49d$^{+}$) (a, c) and CD8α$^{+}$CD11a$^{hi}$CD44$^{+}$) (b, d) gated cells. Error bars, mean ± s.e.m. Data representative of two (M1/NA: n= 3-4 mice/group) or three (Naïve, HA/NP, and NA/NP: n= 4-5 mice/group) independent experiments. * P< 0.05, ** P< 0.01, *** P< 0.001 (One-way ANOVA with Tukey's multiple comparisons test).

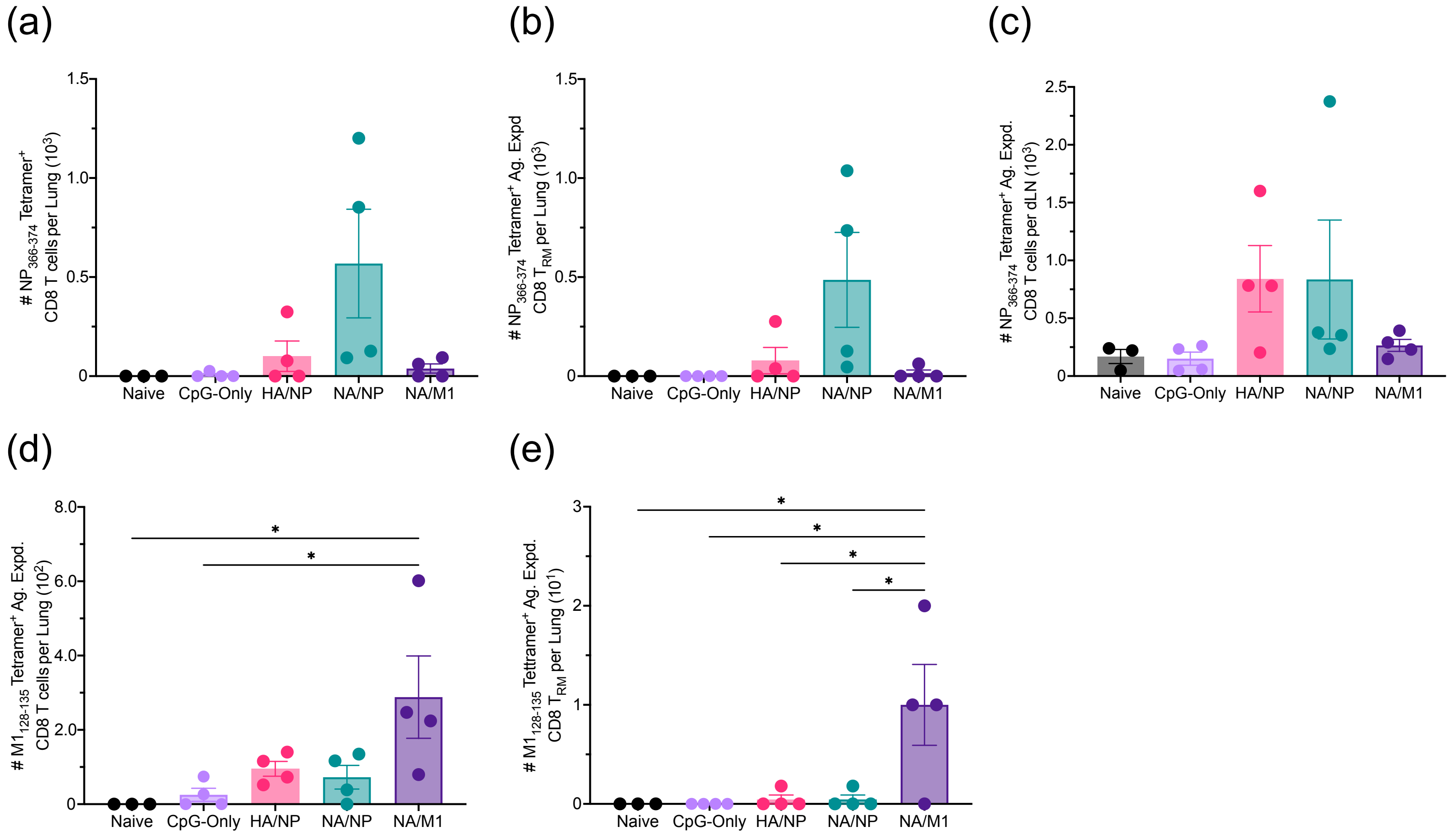


**Supplementary Fig. 10. H3-nanovax formulations generate vaccine specific CD8 T cell responses.** C57BL/6 mice were vaccinated (CpG only-nanovax (light purple), H3-nanovax (HA/NP; Pink), (NA/NP; Blue), (NA/M1; Purple), or left unvaccinated (naïve)) as described in Fig. 2. Lung resident antigen-experienced CD8 ($CD45i.vAb^{-}CD8\alpha^{+}CD44^{+}$) T cells specific for $NP_{366-374}$ and $M1_{128-135}$ derived from IAV A/Hong Kong/1/68 and A/Aichi/2/68 respectively were enumerated (a, b, d, e). Enumeration of dLN antigen experienced ($CD11a^{+}CD44^{+}$) CD8 T cells specific for $NP_{366-374}$ (c). Error bars, mean ± s.e.m. Data representative of one n= 3-4 mice/group) independent experiment. * $P< 0.05$, (One-way ANOVA with Tukey's multiple comparisons test).
