# Supplementary material for: Polyanhydride nanovaccine against H3N2 influenza A virus generates mucosal resident and systemic immunity promoting protection
Source: NPJ Vaccines. 2024 May 31;9:96. doi: 10.1038/s41541-024-00883-3 (PMC11143372; doi:10.1038/s41541-024-00883-3)
Supplement: Supplementary file 2 — Reporting summary [file 41541_2024_883_MOESM2_ESM.pdf]

nature portfolio

Corresponding author(s): Kevin L Legge

Last updated by author(s): Apr 29, 2024

Reporting Summary

Nature Portfolio wishes to improve the reproducibility of the work that we publish. This form provides structure for consistency and transparency in reporting. For further information on Nature Portfolio policies, see our Editorial Policies and the Editorial Policy Checklist.

Statistics

For all statistical analyses, confirm that the following items are present in the figure legend, table legend, main text, or Methods section.

n/a	Confirmed	
☐	☒	The exact sample size (n) for each experimental group/condition, given as a discrete number and unit of measurement
☐	☒	A statement on whether measurements were taken from distinct samples or whether the same sample was measured repeatedly
☐	☒	The statistical test(s) used AND whether they are one- or two-sided<br>*Only common tests should be described solely by name; describe more complex techniques in the Methods section.*
☒	☐	A description of all covariates tested
☐	☒	A description of any assumptions or corrections, such as tests of normality and adjustment for multiple comparisons
☐	☒	A full description of the statistical parameters including central tendency (e.g. means) or other basic estimates (e.g. regression coefficient) AND variation (e.g. standard deviation) or associated estimates of uncertainty (e.g. confidence intervals)
☐	☒	For null hypothesis testing, the test statistic (e.g. F, t, r) with confidence intervals, effect sizes, degrees of freedom and P value noted<br>*Give P values as exact values whenever suitable.*
☒	☐	For Bayesian analysis, information on the choice of priors and Markov chain Monte Carlo settings
☒	☐	For hierarchical and complex designs, identification of the appropriate level for tests and full reporting of outcomes
☒	☐	Estimates of effect sizes (e.g. Cohen's d, Pearson's r), indicating how they were calculated

Our web collection on statistics for biologists contains articles on many of the points above.

Software and code

Policy information about availability of computer code

Data collection	N/A
Data analysis	FlowJo versions 9 and 10 were used to analyze flow cytometry data. Prism version 9 and 10 were used to data graphing and statistical anlsysis

For manuscripts utilizing custom algorithms or software that are central to the research but not yet described in published literature, software must be made available to editors and reviewers. We strongly encourage code deposition in a community repository (e.g. GitHub). See the Nature Portfolio guidelines for submitting code & software for further information.

Data

Policy information about availability of data

All manuscripts must include a data availability statement. This statement should provide the following information, where applicable:

- Accession codes, unique identifiers, or web links for publicly available datasets
- A description of any restrictions on data availability
- For clinical datasets or third party data, please ensure that the statement adheres to our policy

All data generated or analyzed from which conclusions are drawn in this manuscript are available within the paper and Supplementary Information.

Research involving human participants, their data, or biological material

Policy information about studies with human participants or human data. See also policy information about sex, gender (identity/presentation), and sexual orientation and race, ethnicity and racism.

Reporting on sex and gender	N/A. no human samples or material
Reporting on race, ethnicity, or other socially relevant groupings	N/A. no human samples or material
Population characteristics	N/A. no human samples or material
Recruitment	N/A. no human samples or material
Ethics oversight	N/A. no human samples or material

Note that full information on the approval of the study protocol must also be provided in the manuscript.

Field-specific reporting

Please select the one below that is the best fit for your research. If you are not sure, read the appropriate sections before making your selection.

☒ Life sciences ☐ Behavioural & social sciences ☐ Ecological, evolutionary & environmental sciences

For a reference copy of the document with all sections, see nature.com/documents/nr-reporting-summary-flat.pdf

Life sciences study design

All studies must disclose on these points even when the disclosure is negative.

Sample size	Sample sizes were not predetermined based on specific statistical methods. The number of animals per group in the study generated data giving sufficient statistics for the effect sizes of interest.
Data exclusions	No data are excluded
Replication	For biological replicates, 3-5 mice were randomly assigned to each group and experiments were performed at least twice
Randomization	Animals were randomly assigned to groups in the study
Blinding	Investigators were not blinded to the study. Blinding is not typically necessary in this type of study

Reporting for specific materials, systems and methods

We require information from authors about some types of materials, experimental systems and methods used in many studies. Here, indicate whether each material, system or method listed is relevant to your study. If you are not sure if a list item applies to your research, read the appropriate section before selecting a response.

Materials & experimental systems

n/a	Involved in the study	
☐	☒	Antibodies
☒	☐	Eukaryotic cell lines
☒	☐	Palaeontology and archaeology
☐	☒	Animals and other organisms
☒	☐	Clinical data
☒	☐	Dual use research of concern
☒	☐	Plants

Methods

n/a	Involved in the study	
☒	☐	ChIP-seq
☐	☒	Flow cytometry
☒	☐	MRI-based neuroimaging

Antibodies

Antibodies used	Antibodies: hamster anti-mouse KLRG1 (2F1; BD Biosciences), rat anti-mouse CD62L (MEL-14; Biolegend), rat anti-mouse CD4 (GK1.5; BioLegend), rat anti-mouse CD8α (53-6.7; BioLegend), rat anti-mouse CD49d (R1-2, BioLegend), rat anti-mouse CD11a (M17/4; BD Biosciences, San Jose, CA), rat anti-mouse CD103 (M290; BD Biosciences), and rat anti-mouse CD69 (H1.2F3; eBioscience), rat anti-

	mouse CD19 (1D3; BD Biosciences), rat anti-mouse B220 (RA3-6B2; BioLegend), rat anti-mouse IgM (B7-6), rat anti-mouse Fas (Jo2; BioLegend), and rat anti-mouse GL7 (GL7; Biolegend), rat anti-mouse CD38 (90; Biolegend), hamster anti-mouse CD69 (H1.2F3; Biolegend), rat anti-mouse CD73 (TY/11.8; Biolegend), and hamster anti-mouse CXCR3 (CXCR3-173; Biolegend), H2-Db NP366-374 tetramer, H2-Kb M1128-135 tetramer; rat anti-mouse Eomes (Dan11mag; Invitrogen), hamster anti-mouse/rat CD49a (Ha31/8; BD Biosciences), hamster anti-mouse CXCR3 (CXCR3-173; Biolegend), and anti-mouse CX3CR1 (SA011F11; Biolegend)
Validation	Validation was performed by vendors. Antibodies were titrated to determine optimal staining concentrations.

Animals and other research organisms

Policy information about studies involving animals; ARRIVE guidelines recommended for reporting animal research, and Sex and Gender in Research

Laboratory animals	Female 8–12-week-old C57Bl/6 mice purchased from Charles River Laboratories
Wild animals	N/A
Reporting on sex	Adult female mice were used
Field-collected samples	N/A
Ethics oversight	The animal protocol was approved by the Institutional Animal Care and Use Committee of the University of Iowa and comply with the NIH Guide for Care and Use of Laboratory Animals

Note that full information on the approval of the study protocol must also be provided in the manuscript.

Plants

Seed stocks	N/A
Novel plant genotypes	N/A
Authentication	N/A

Flow Cytometry

Plots

Confirm that:

☒ The axis labels state the marker and fluorochrome used (e.g. CD4-FITC).

☒ The axis scales are clearly visible. Include numbers along axes only for bottom left plot of group (a 'group' is an analysis of identical markers).

☒ All plots are contour plots with outliers or pseudocolor plots.

☒ A numerical value for number of cells or percentage (with statistics) is provided.

Methodology

Sample preparation	- blood was collected in non-heparinized capillary tubes for serum collection. Blood samples were left at room temperature for 30 min, centrifuged at 16,000 × g for 20 min, and then collected and stored at −20°C until analysis.<br>- Bronchial alveolar lavage (BAL) fluid was collected using a protocol modified from. Briefly, the tracheae were cannulated with a 22-gage catheter tube (attached to a 5cc syringe) and then washed once with 1 mL of sterile PBS. Samples were stored at −20°C until analysis<br>- lungs and mediastinal lymph nodes were harvested, digested, homogenized in gentleMACS™ C tubes utilizing the gentleMACS™ Octo Dissociator and subsequently strained through 70 μm filters into single cell suspensions
Instrument	Data were acquired on a BD LSRII (BD Biosciences) or Cytek Aurora (Cytek Biosciences)
Software	Flow cytometry data was collected using FACSDiva or Spectroflo software and analyzed in FlowJo 9 and 10
Cell population abundance	No cell sorting was performed in this study
Gating strategy	Live (Viability dye), single cell (FSC-H vs FSC-A) lymphocytes (FSC-A vs SSC-A) were gated first. Boundaries between positive

Gating strategy	and negative populations were defined based on FMO controls and populations of cells definitively known to possess or lack expression of the marker of interest.

☒ Tick this box to confirm that a figure exemplifying the gating strategy is provided in the Supplementary Information.
